# Supplementary material for: Genetic architecture of complex traits and disease risk predictors
Source: Sci Rep. 2020 Jul 21;10:12055. doi: 10.1038/s41598-020-68881-8 (PMC7374622; doi:10.1038/s41598-020-68881-8)
Supplement: Supplementary file 1 — Supplementary Information 1. [file 41598_2020_68881_MOESM1_ESM.pdf]

Genetic Architecture of Complex Traits and Disease Risk Predictors

Soke Yuen Yong *[1], Timothy G. Raben †[1], Louis Lello ‡[1,2], and Stephen D.H. Hsu §[1,2]

[1]Department of Physics and Astronomy, Michigan State University
[2]Genomic Prediction, North Brunswick, NJ

Supplementary Information

Appendix A: Supplementary Figures

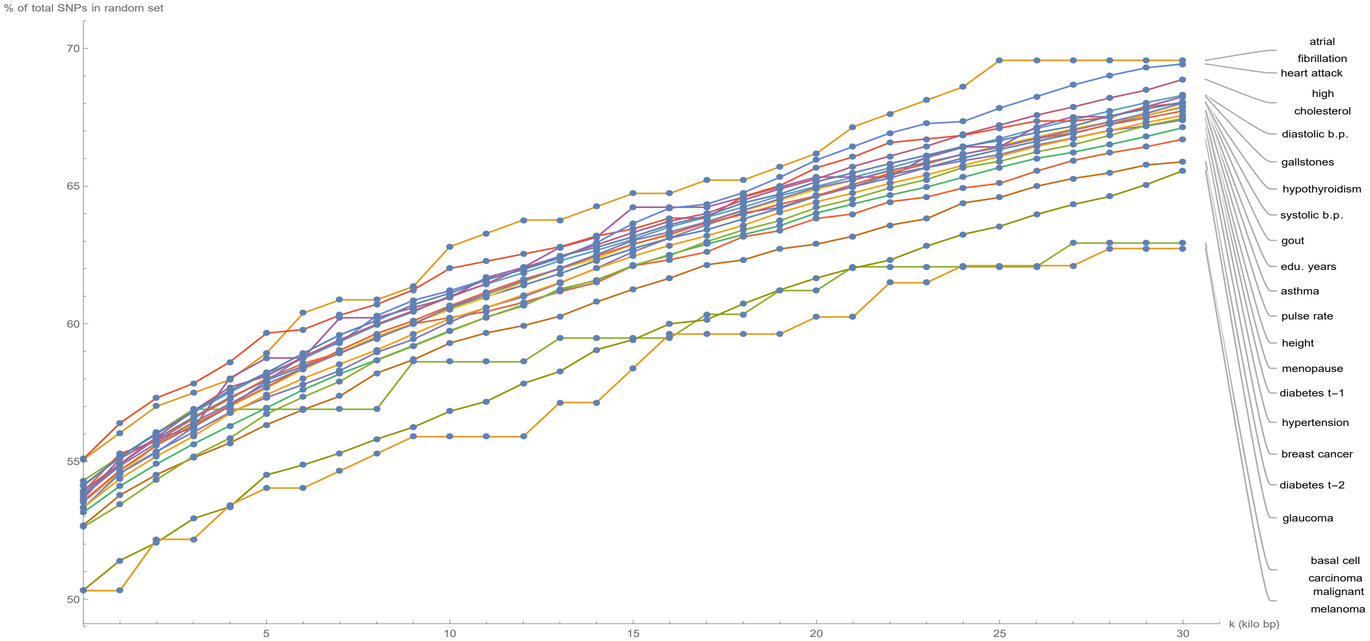

Supplementary Figure S1: *Plots of the number of random SNPs located within genic regions, expressed as a percentage of the total number of SNPs in a randomly-selected set containing the same number of SNPs as the activated set in the predictor for the indicated disease condition/trait, against expansion of GENCODE Release 19 gene boundaries by k kilo base pairs. SNPS were randomly selected from the 800,000+ variants measured by the UK Biobank Axiom Array.*

*Corresponding Author: yongsoke@msu.edu
†rabentim@msu.edu
‡lellolou@msu.edu
§hsu@msu.edu

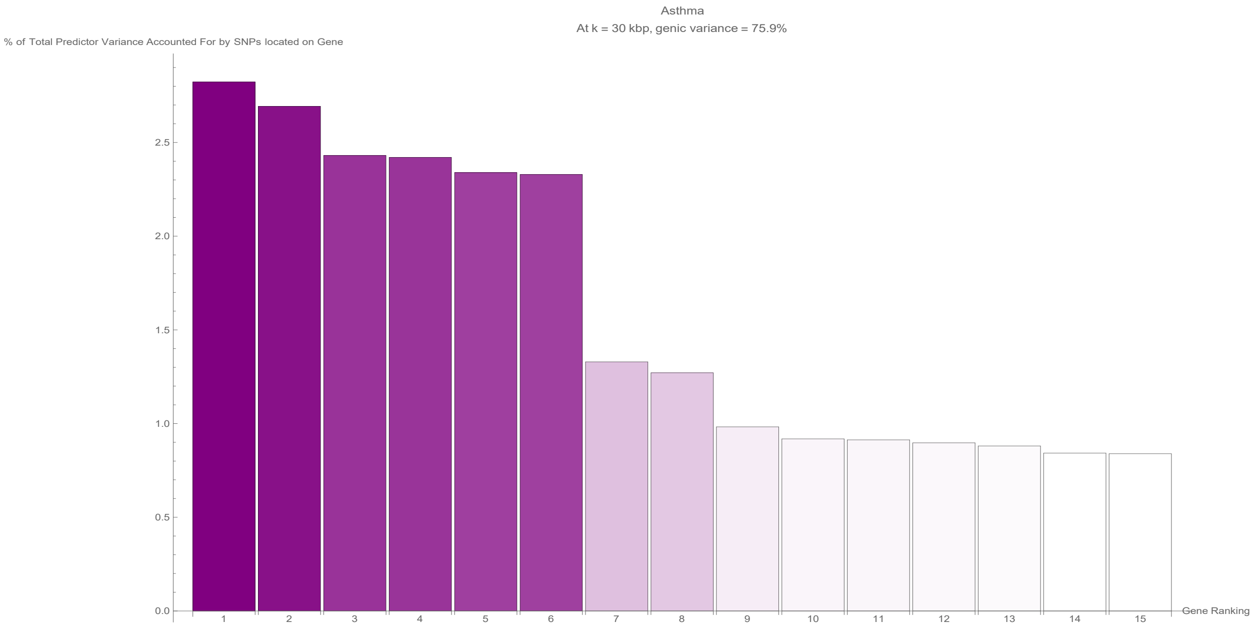

Supplementary Figure S2: *The fifteen largest total values of variance accounted for by predictor SNPs located on a single gene (in terms of the percentage of total variance accounted for by all predictor SNPs) for the asthma predictor. Each vertical bar is colored violet with a depth of shade proportional to the height of the bar. Here, 'genic' SNPs are contained within the GENCODE Release 19 gene boundaries plus 30 kilo base pairs at both ends.*

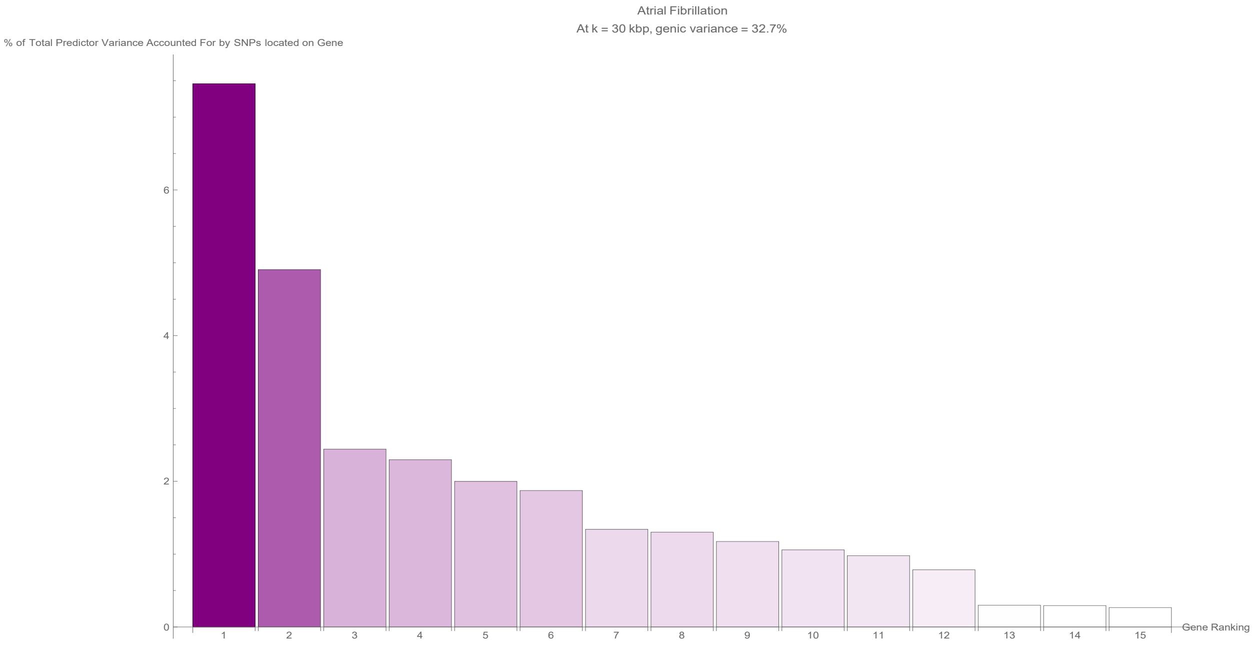

Supplementary Figure S3: *The fifteen largest total values of variance accounted for by predictor SNPs located on a single gene (in terms of the percentage of total variance accounted for by all predictor SNPs) for the atrial fibrillation predictor. Each vertical bar is colored violet with a depth of shade proportional to the height of the bar. Here, 'genic' SNPs are contained within the GENCODE Release 19 gene boundaries plus 30 kilo base pairs at both ends.*

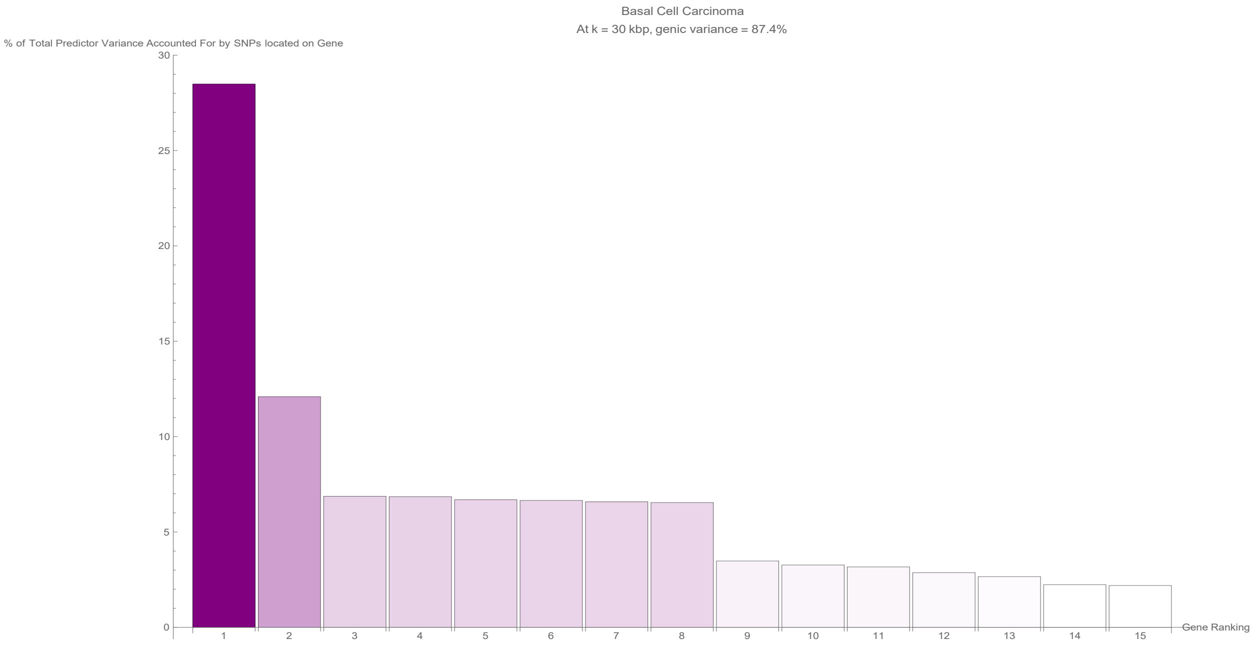

Supplementary Figure S4: *The fifteen largest total values of variance accounted for by predictor SNPs located on a single gene (in terms of the percentage of total variance accounted for by all predictor SNPs) for the basal cell carcinoma predictor. Each vertical bar is colored violet with a depth of shade proportional to the height of the bar. Here, 'genic' SNPs are contained within the GENCODE Release 19 gene boundaries plus 30 kilo base pairs at both ends.*

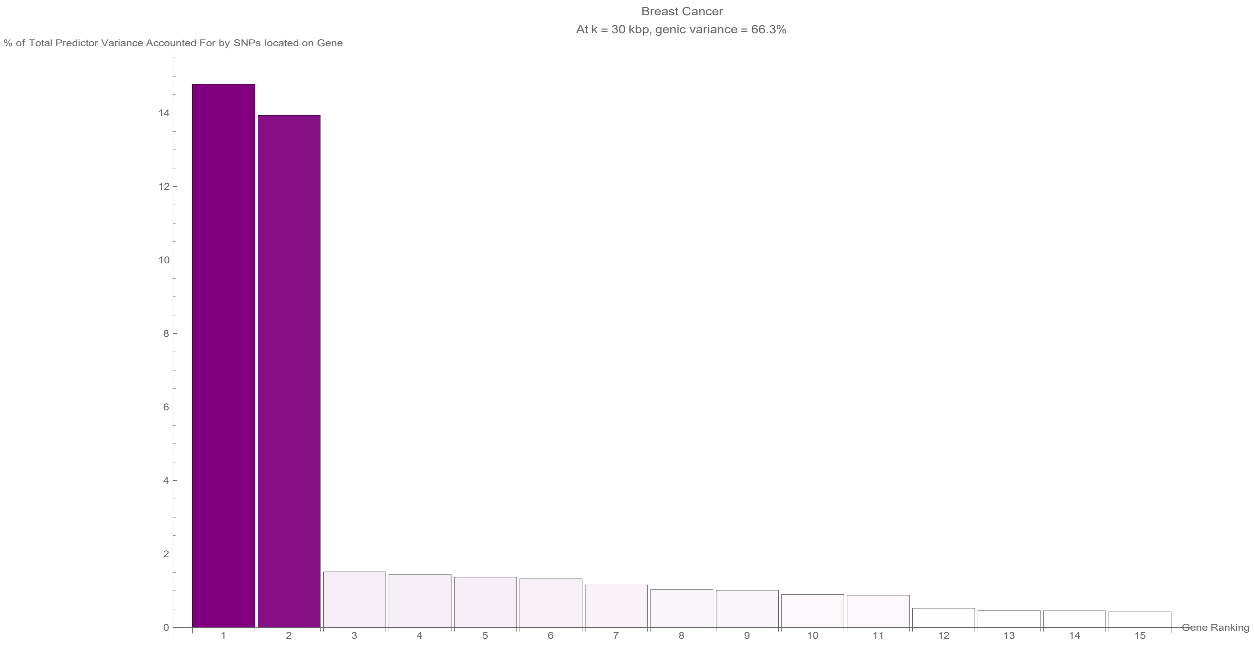

Supplementary Figure S5: *The fifteen largest total values of variance accounted for by predictor SNPs located on a single gene (in terms of the percentage of total variance accounted for by all predictor SNPs) for the breast cancer predictor. Each vertical bar is colored violet with a depth of shade proportional to the height of the bar. Here, 'genic' SNPs are contained within the GENCODE Release 19 gene boundaries plus 30 kilo base pairs at both ends.*

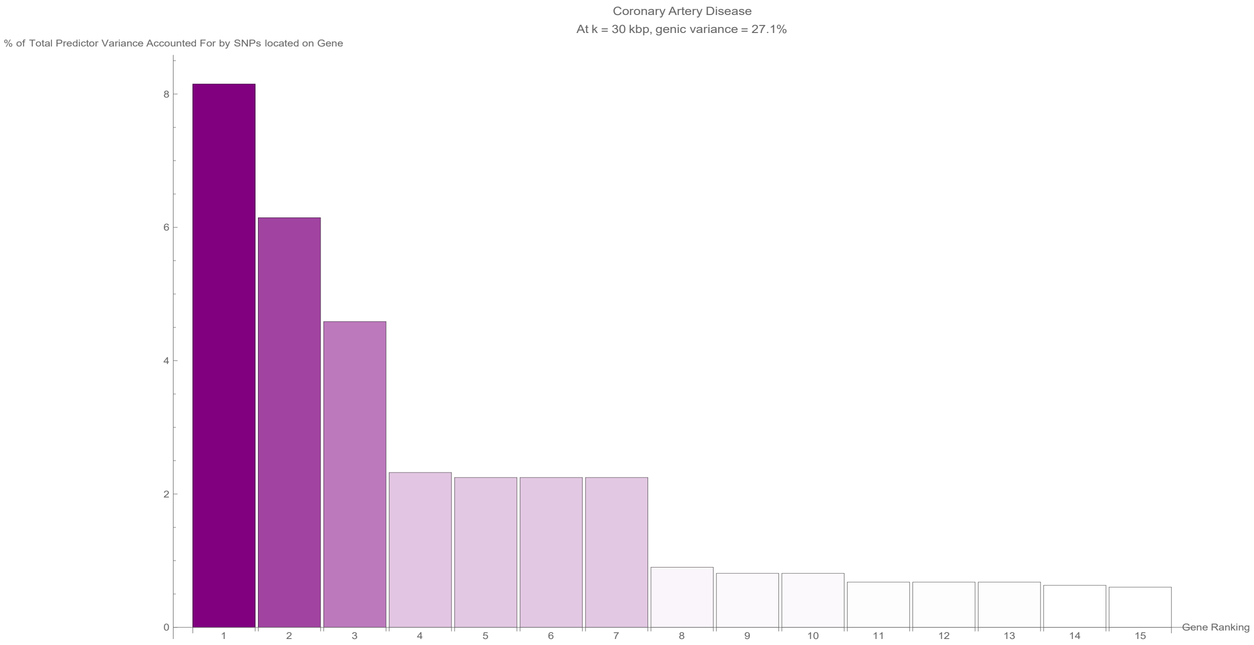

Supplementary Figure S6: *The fifteen largest total values of variance accounted for by predictor SNPs located on a single gene (in terms of the percentage of total variance accounted for by all predictor SNPs) for the coronary artery disease predictor. Each vertical bar is colored violet with a depth of shade proportional to the height of the bar. Here, 'genic' SNPs are contained within the GENCODE Release 19 gene boundaries plus 30 kilo base pairs at both ends.*

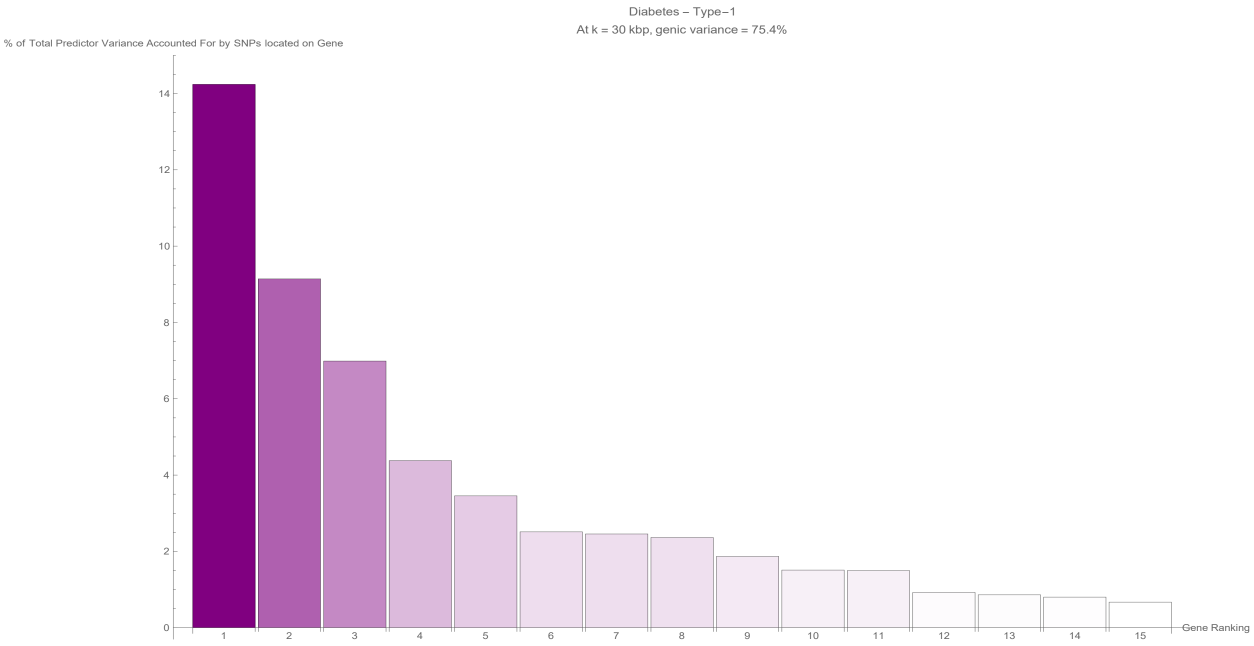

Supplementary Figure S7: *The fifteen largest total values of variance accounted for by predictor SNPs located on a single gene (in terms of the percentage of total variance accounted for by all predictor SNPs) for the type-1 diabetes predictor. Each vertical bar is colored violet with a depth of shade proportional to the height of the bar. Here, 'genic' SNPs are contained within the GENCODE Release 19 gene boundaries plus 30 kilo base pairs at both ends.*

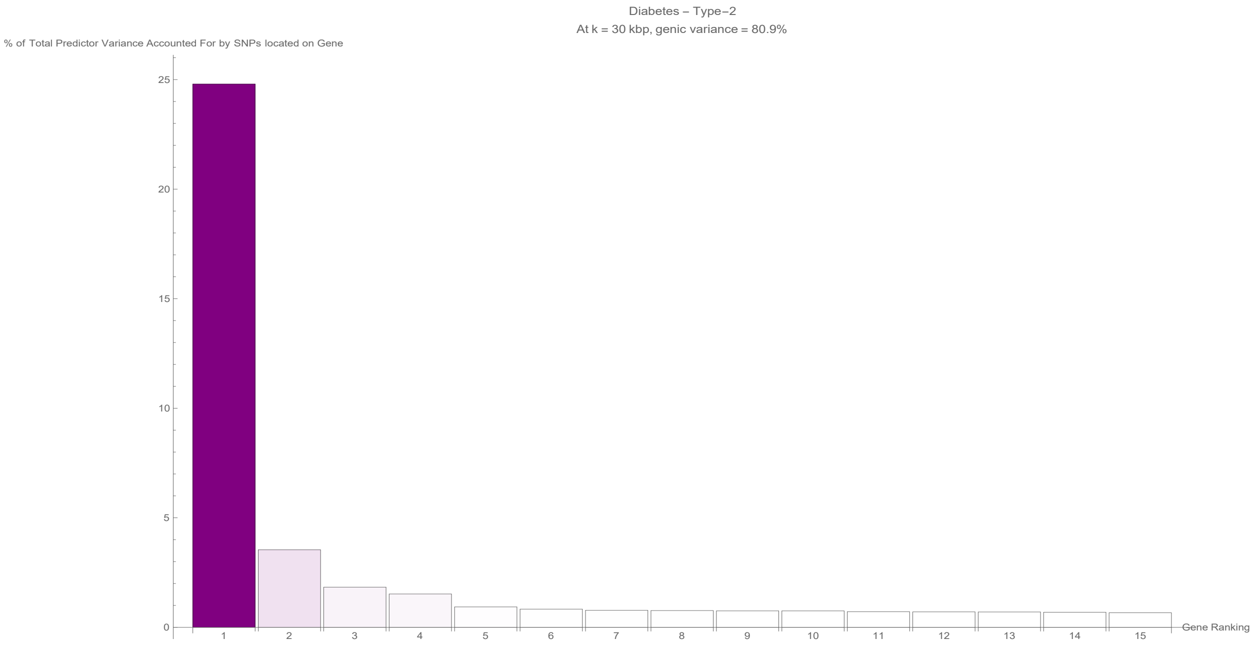

Supplementary Figure S8: *The fifteen largest total values of variance accounted for by predictor SNPs located on a single gene (in terms of the percentage of total variance accounted for by all predictor SNPs) for the type-2 diabetes predictor. Each vertical bar is colored violet with a depth of shade proportional to the height of the bar. Here, 'genic' SNPs are contained within the GENCODE Release 19 gene boundaries plus 30 kilo base pairs at both ends.*

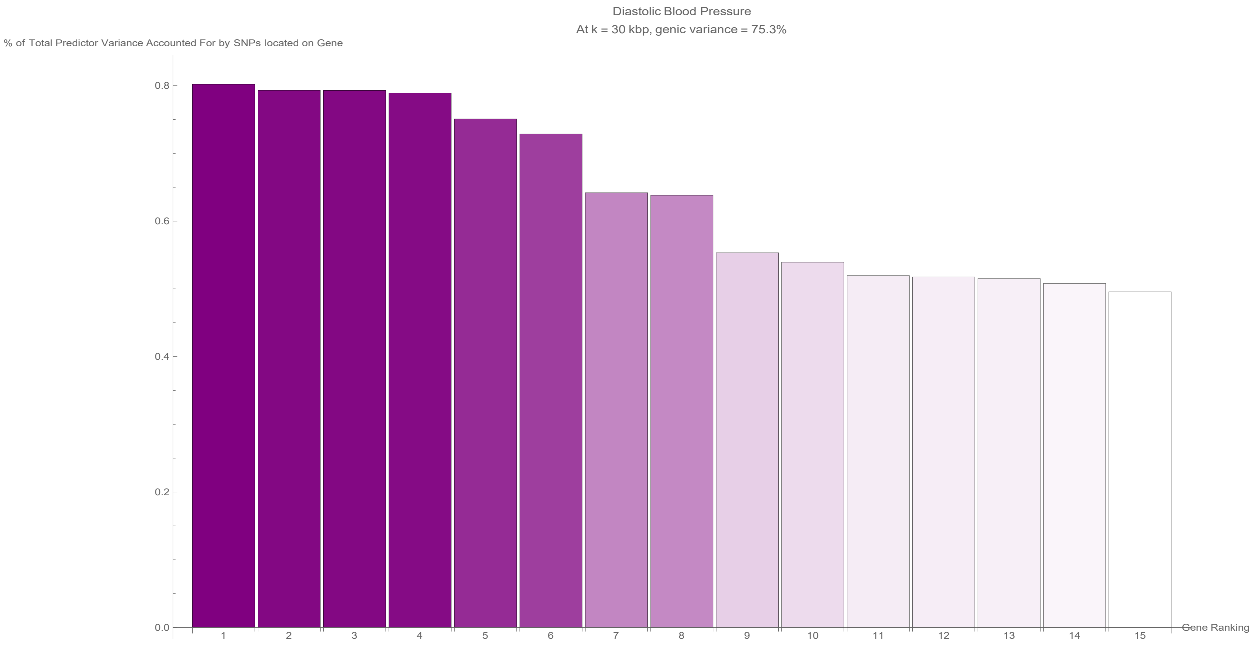

Supplementary Figure S9: *The fifteen largest total values of variance accounted for by predictor SNPs located on a single gene (in terms of the percentage of total variance accounted for by all predictor SNPs) for the diastolic blood pressure predictor. Each vertical bar is colored violet with a depth of shade proportional to the height of the bar. Here, 'genic' SNPs are contained within the GENCODE Release 19 gene boundaries plus 30 kilo base pairs at both ends.*

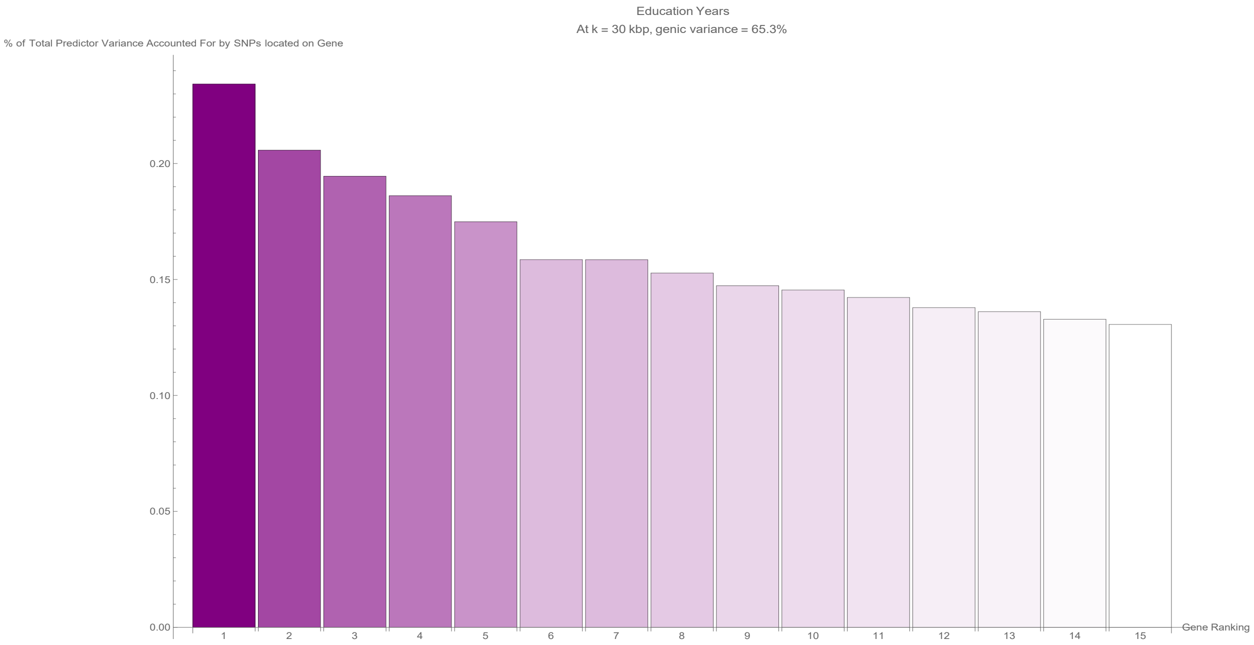

Supplementary Figure S10: *The fifteen largest total values of variance accounted for by predictor SNPs located on a single gene (in terms of the percentage of total variance accounted for by all predictor SNPs) for the education years predictor. Each vertical bar is colored violet with a depth of shade proportional to the height of the bar. Here, 'genic' SNPs are contained within the GENCODE Release 19 gene boundaries plus 30 kilo base pairs at both ends.*

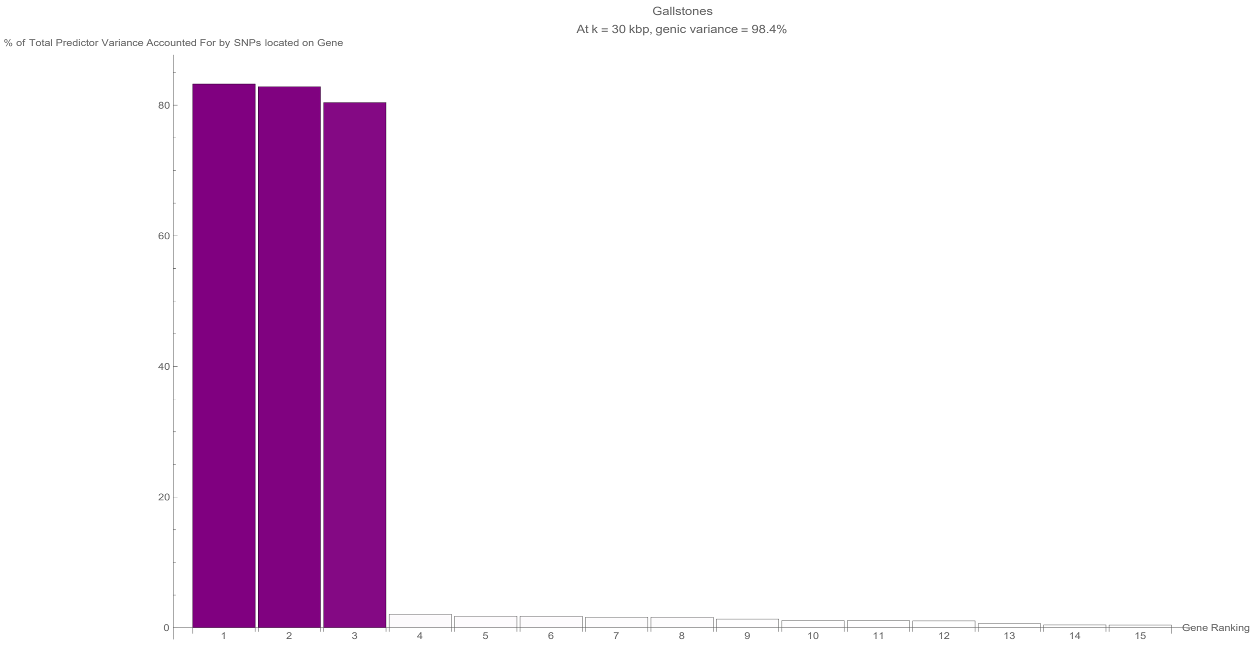

Supplementary Figure S11: *The fifteen largest total values of variance accounted for by predictor SNPs located on a single gene (in terms of the percentage of total variance accounted for by all predictor SNPs) for the gallstones predictor. Each vertical bar is colored violet with a depth of shade proportional to the height of the bar. Here, 'genic' SNPs are contained within the GENCODE Release 19 gene boundaries plus 30 kilo base pairs at both ends.*

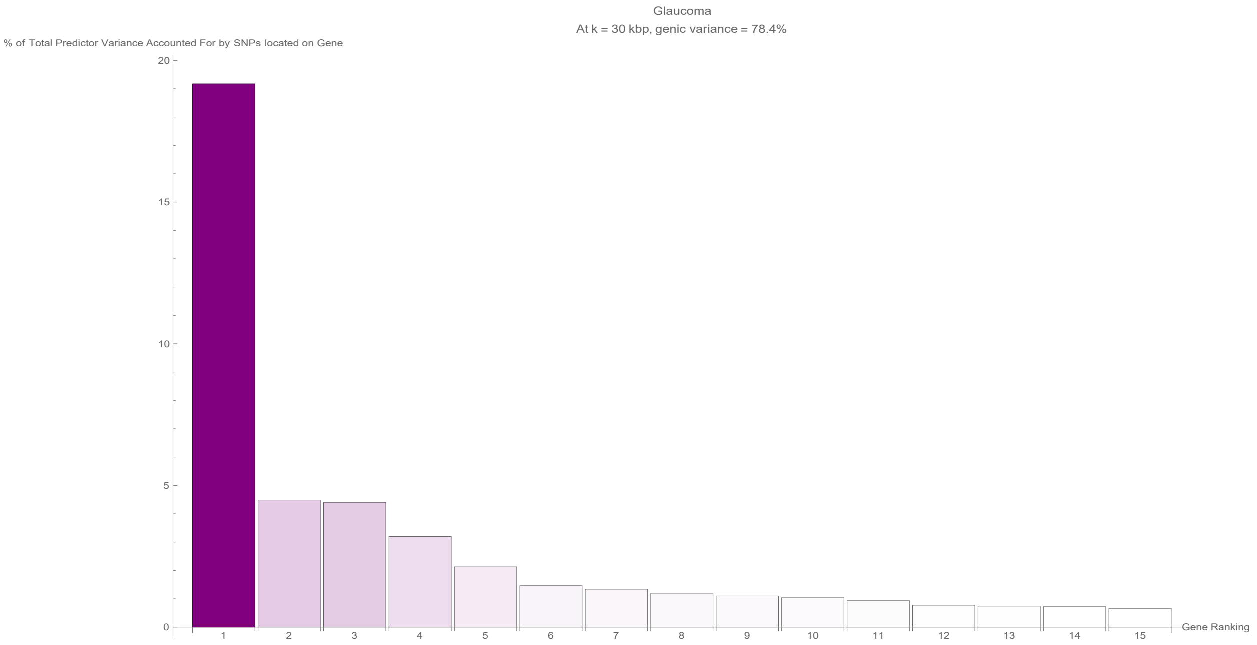

Supplementary Figure S12: *The fifteen largest total values of variance accounted for by predictor SNPs located on a single gene (in terms of the percentage of total variance accounted for by all predictor SNPs) for the glaucoma predictor. Each vertical bar is colored violet with a depth of shade proportional to the height of the bar. Here, 'genic' SNPs are contained within the GENCODE Release 19 gene boundaries plus 30 kilo base pairs at both ends.*

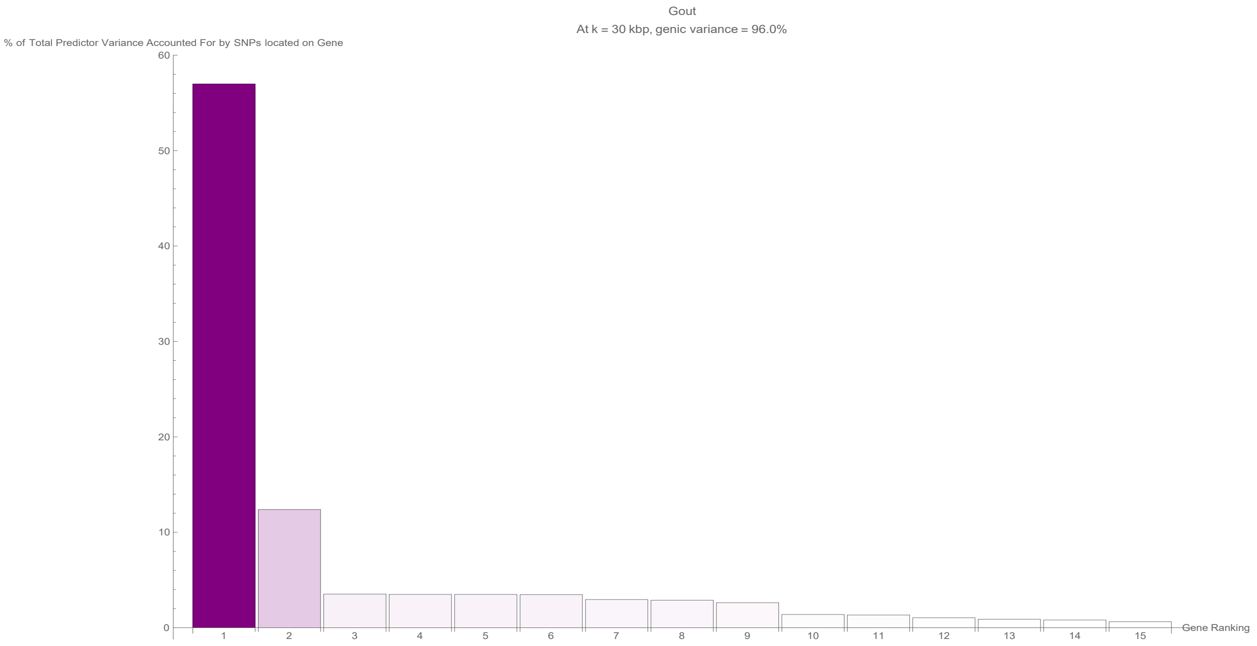

Supplementary Figure S13: *The fifteen largest total values of variance accounted for by predictor SNPs located on a single gene (in terms of the percentage of total variance accounted for by all predictor SNPs) for the gout predictor. Each vertical bar is colored violet with a depth of shade proportional to the height of the bar. Here, 'genic' SNPs are contained within the GENCODE Release 19 gene boundaries plus 30 kilo base pairs at both ends.*

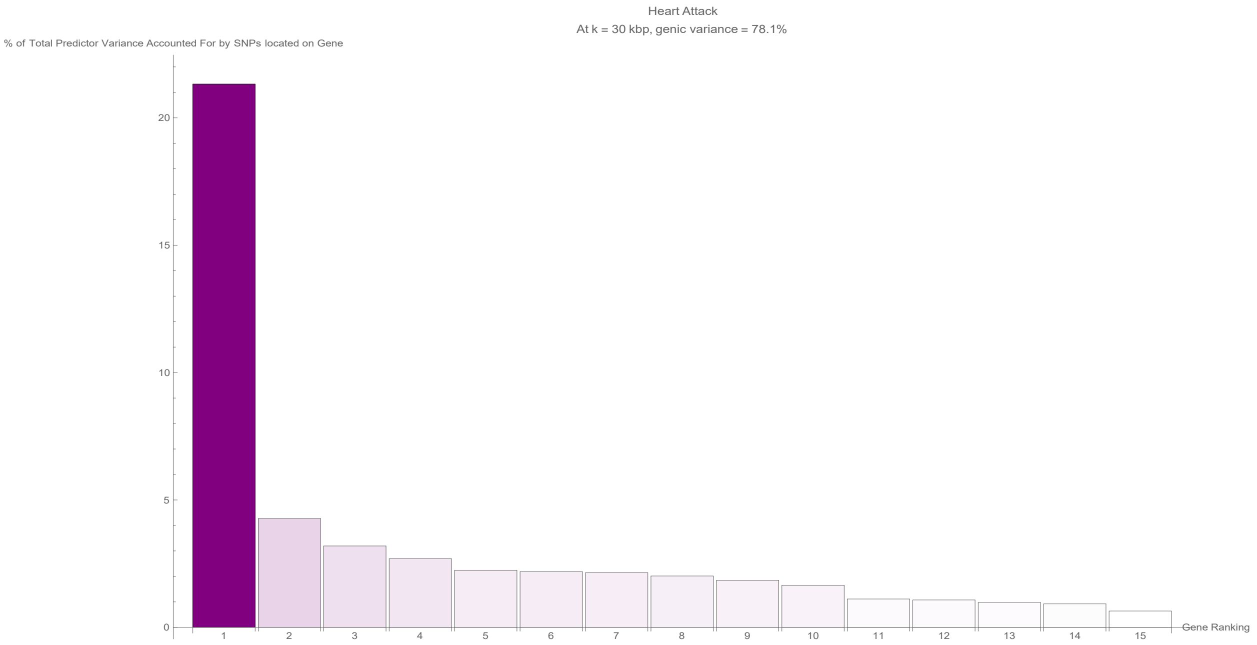

Supplementary Figure S14: *The fifteen largest total values of variance accounted for by predictor SNPs located on a single gene (in terms of the percentage of total variance accounted for by all predictor SNPs) for the heart attack predictor. Each vertical bar is colored violet with a depth of shade proportional to the height of the bar. Here, 'genic' SNPs are contained within the GENCODE Release 19 gene boundaries plus 30 kilo base pairs at both ends.*

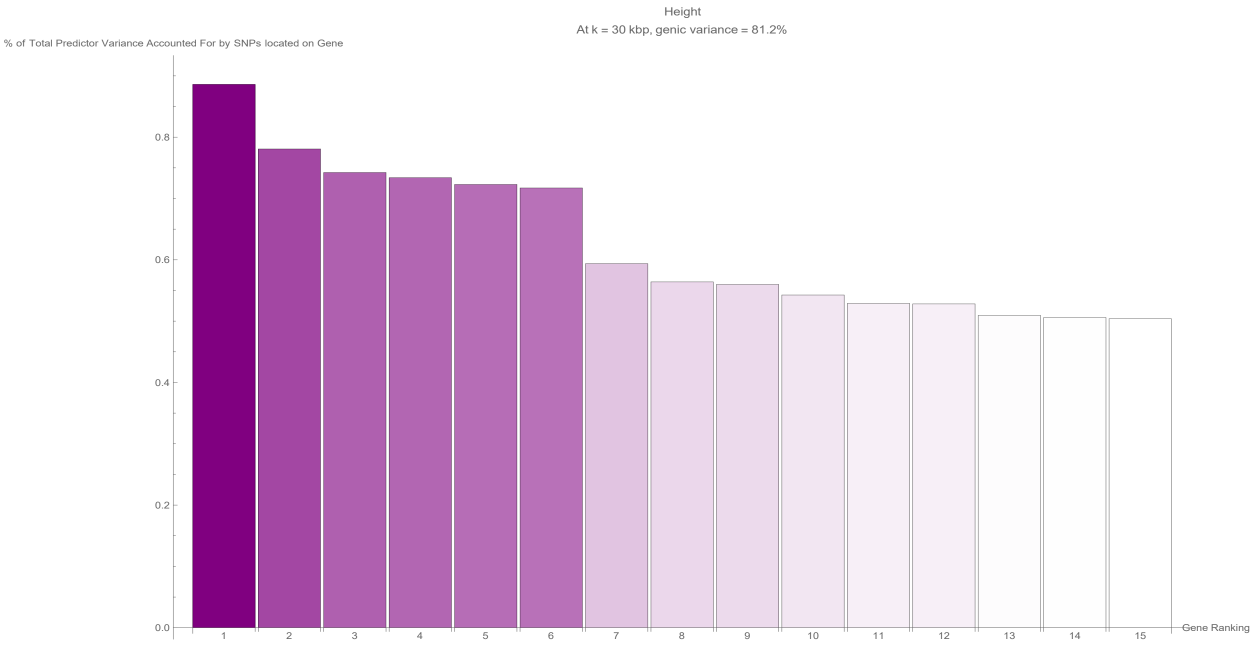

Supplementary Figure S15: *The fifteen largest total values of variance accounted for by predictor SNPs located on a single gene (in terms of the percentage of total variance accounted for by all predictor SNPs) for the height predictor. Each vertical bar is colored violet with a depth of shade proportional to the height of the bar. Here, 'genic' SNPs are contained within the GENCODE Release 19 gene boundaries plus 30 kilo base pairs at both ends.*

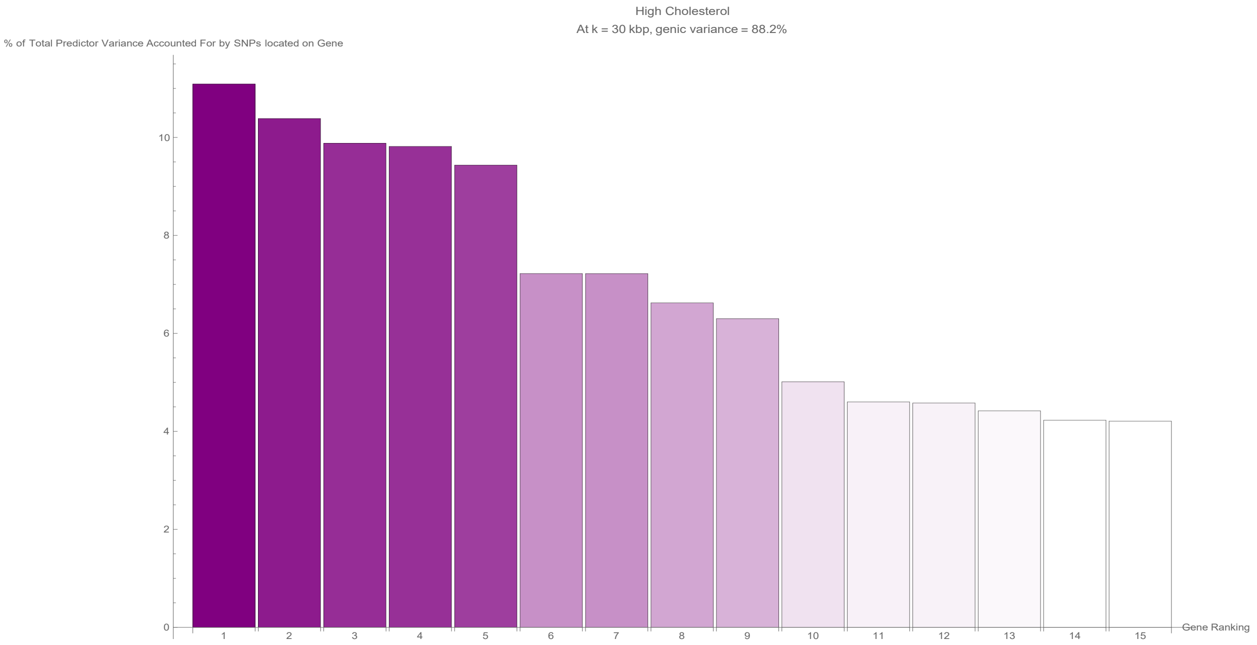

Supplementary Figure S16: *The fifteen largest total values of variance accounted for by predictor SNPs located on a single gene (in terms of the percentage of total variance accounted for by all predictor SNPs) for the high cholesterol predictor. Each vertical bar is colored violet with a depth of shade proportional to the height of the bar. Here, 'genic' SNPs are contained within the GENCODE Release 19 gene boundaries plus 30 kilo base pairs at both ends.*

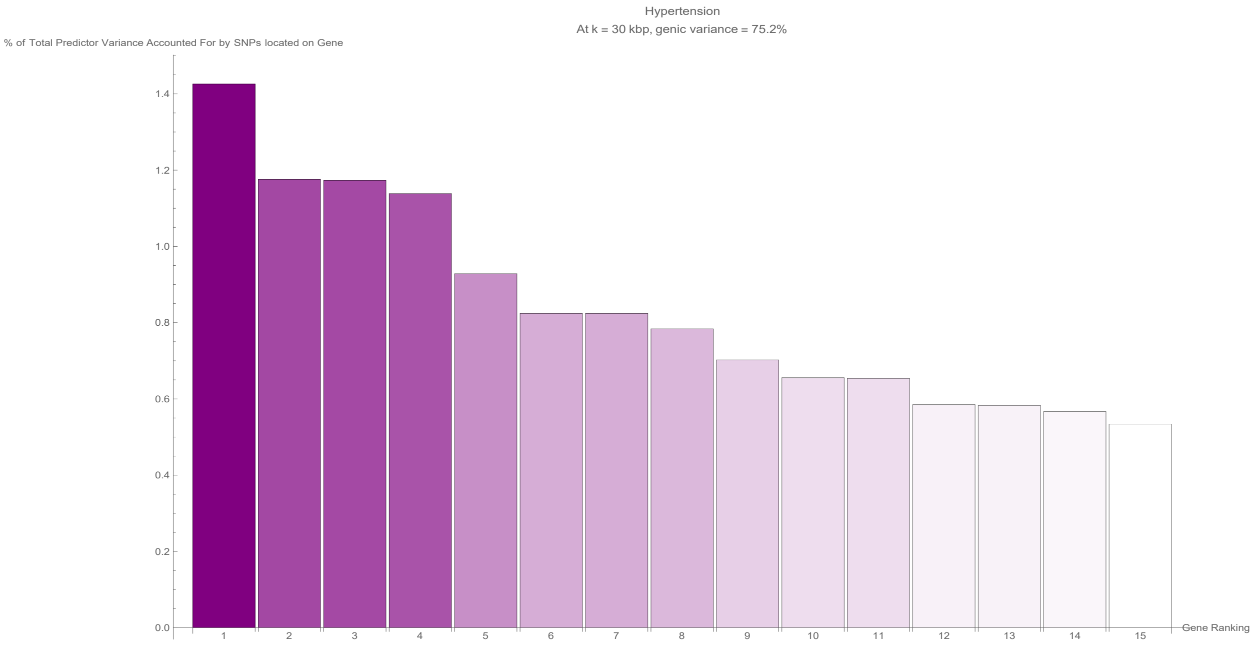

Supplementary Figure S17: *The fifteen largest total values of variance accounted for by predictor SNPs located on a single gene (in terms of the percentage of total variance accounted for by all predictor SNPs) for the hypertension predictor. Each vertical bar is colored violet with a depth of shade proportional to the height of the bar. Here, 'genic' SNPs are contained within the GENCODE Release 19 gene boundaries plus 30 kilo base pairs at both ends.*

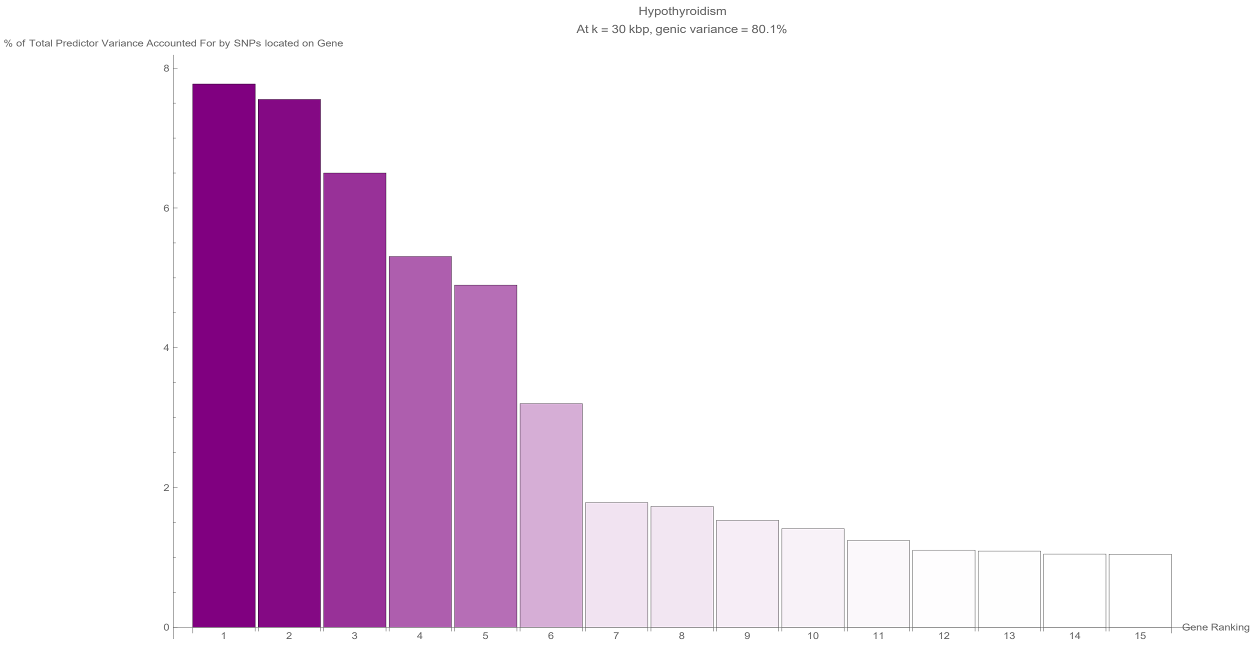

Supplementary Figure S18: *The fifteen largest total values of variance accounted for by predictor SNPs located on a single gene (in terms of the percentage of total variance accounted for by all predictor SNPs) for the hypothyroidism predictor. Each vertical bar is colored violet with a depth of shade proportional to the height of the bar. Here, 'genic' SNPs are contained within the GENCODE Release 19 gene boundaries plus 30 kilo base pairs at both ends.*

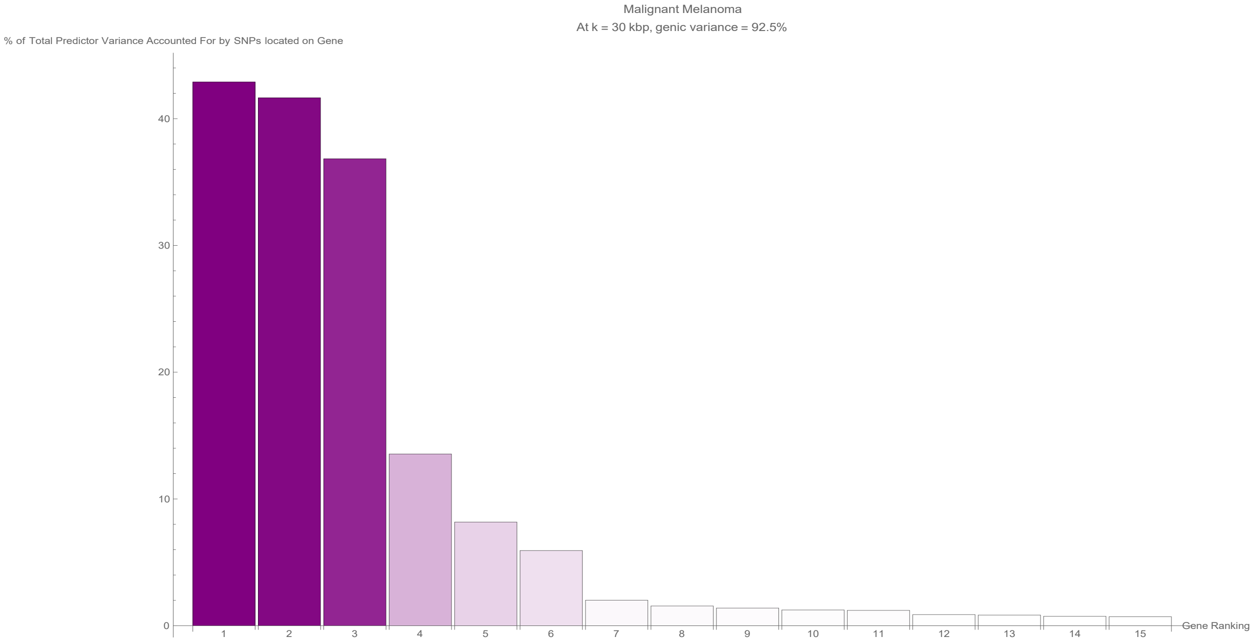

Supplementary Figure S19: *The fifteen largest total values of variance accounted for by predictor SNPs located on a single gene (in terms of the percentage of total variance accounted for by all predictor SNPs) for the malignant melanoma predictor. Each vertical bar is colored violet with a depth of shade proportional to the height of the bar. Here, 'genic' SNPs are contained within the GENCODE Release 19 gene boundaries plus 30 kilo base pairs at both ends.*

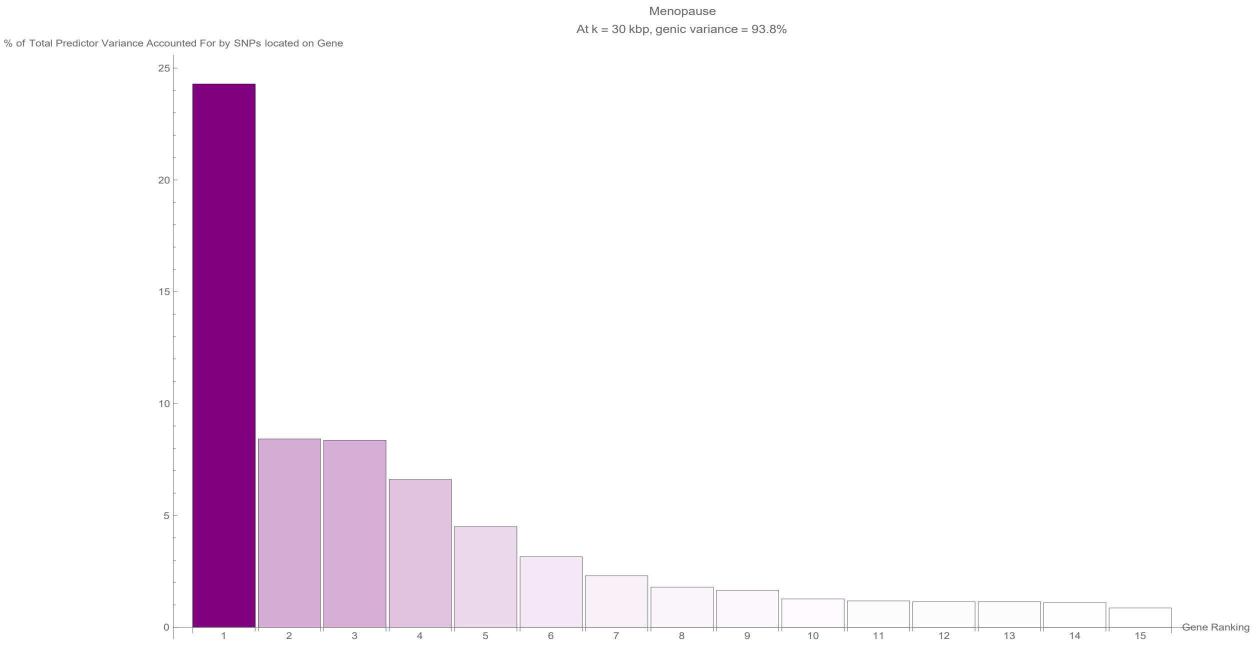

Supplementary Figure S20: *The fifteen largest total values of variance accounted for by predictor SNPs located on a single gene (in terms of the percentage of total variance accounted for by all predictor SNPs) for the menopause predictor. Each vertical bar is colored violet with a depth of shade proportional to the height of the bar. Here, 'genic' SNPs are contained within the GENCODE Release 19 gene boundaries plus 30 kilo base pairs at both ends.*

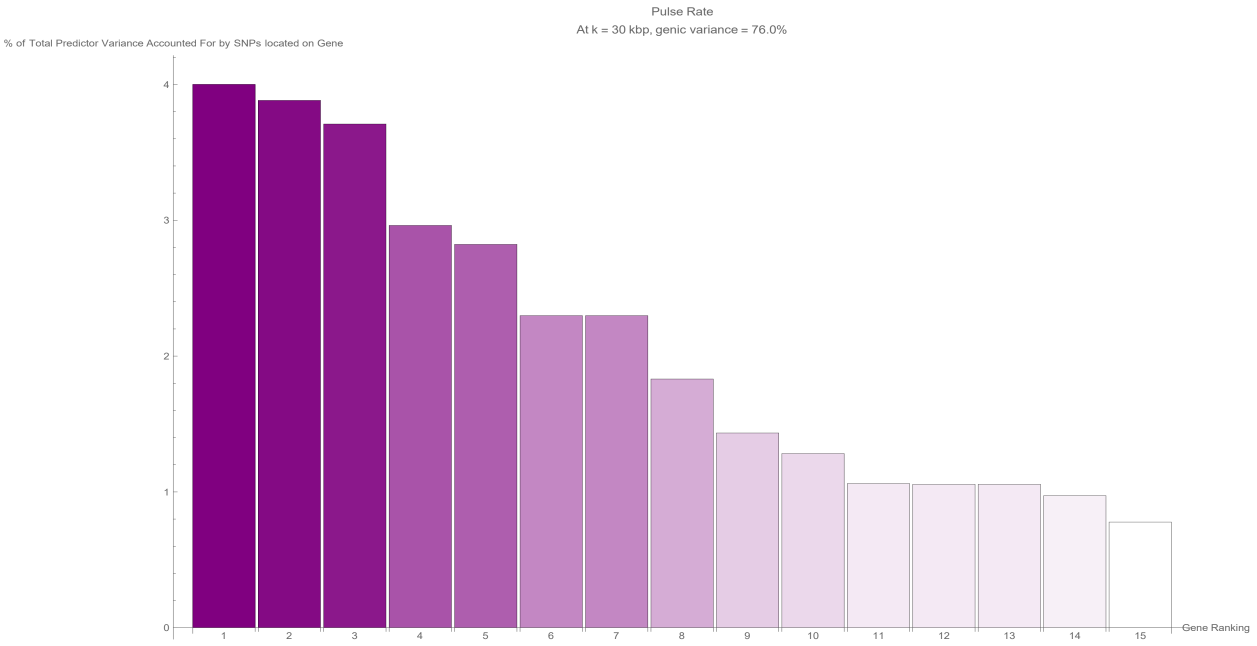

Supplementary Figure S21: *The fifteen largest total values of variance accounted for by predictor SNPs located on a single gene (in terms of the percentage of total variance accounted for by all predictor SNPs) for the pulse rate predictor. Each vertical bar is colored violet with a depth of shade proportional to the height of the bar. Here, 'genic' SNPs are contained within the GENCODE Release 19 gene boundaries plus 30 kilo base pairs at both ends.*

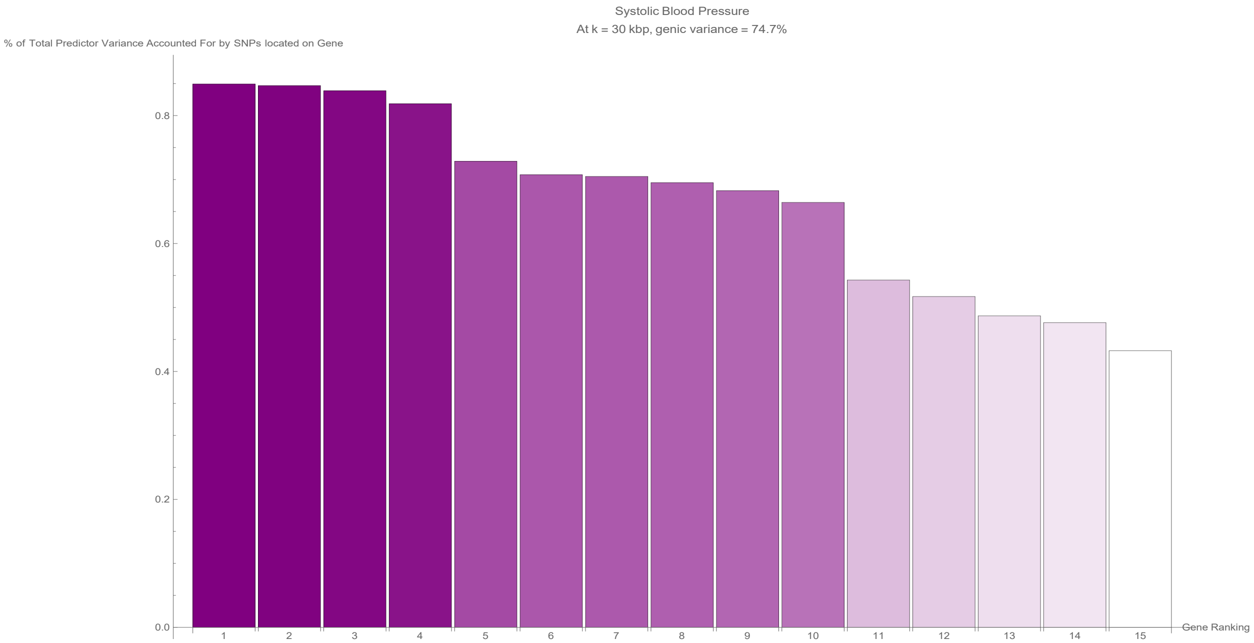

Supplementary Figure S22: *The fifteen largest total values of variance accounted for by predictor SNPs located on a single gene (in terms of the percentage of total variance accounted for by all predictor SNPs) for the systolic blood pressure predictor. Each vertical bar is colored violet with a depth of shade proportional to the height of the bar. Here, 'genic' SNPs are contained within the GENCODE Release 19 gene boundaries plus 30 kilo base pairs at both ends.*

Appendix B: Supplementary Tables

Tables that list the top genes, as ordered by variance accounted for, for various phenotypes.

Asthma

Ensembl ID	Variance Explained by Gene	% of Total Predictor Variance
ENSG00000196735	0.0000461821	2.82293
ENSG00000166949	0.0000440566	2.69301
ENSG00000115604	0.000039764	2.43062
ENSG00000115602	0.0000395971	2.42042
ENSG00000145777	0.0000382693	2.33925
ENSG00000134987	0.0000382693	2.33925
ENSG00000179344	0.0000381038	2.32914
ENSG00000137033	0.0000217498	1.32948
ENSG00000038532	0.0000207941	1.27106
ENSG00000113522	0.0000160743	0.982561
ENSG00000073605	0.0000150246	0.918398
ENSG00000172057	0.0000150246	0.918398
ENSG00000166888	0.0000149384	0.913124
ENSG00000166881	0.0000146788	0.897259
ENSG00000166886	0.0000146788	0.897259
ENSG00000186075	0.0000144062	0.880593
ENSG00000145012	0.0000137849	0.84262
ENSG00000158636	0.0000137277	0.839121

Supplementary Table S23: *For the asthma predictor: list of genes responsible for the top fifteen values of variance accounted for by single genes, and the corresponding variance values, both explicit and expressed as a percentage of the total predictor variance.*

Atrial Fibrillation

Ensembl ID	Variance Explained by Gene	% of Total Predictor Variance
ENSG00000140836	4.35491×10^{-7}	7.45884
ENSG00000164093	2.86504×10^{-7}	4.90708
ENSG00000116132	1.42571×10^{-7}	2.44188
ENSG00000143603	1.34099×10^{-7}	2.29676
ENSG00000107954	1.16675×10^{-7}	1.99835
ENSG00000148120	1.09383×10^{-7}	1.87345
ENSG00000089225	7.82504×10^{-8}	1.34023
ENSG00000115935	7.60088×10^{-8}	1.30184
ENSG00000107957	6.85145×10^{-8}	1.17348
ENSG00000105974	6.17914×10^{-8}	1.05833
ENSG00000171385	5.7165×10^{-8}	0.97909
ENSG00000173406	4.58225×10^{-8}	0.784823
ENSG00000110318	1.74445×10^{-8}	0.298779
ENSG00000120457	1.71183×10^{-8}	0.293193
ENSG00000174370	1.71183×10^{-8}	0.293193
ENSG00000173572	1.55386×10^{-8}	0.266137
ENSG00000179709	1.55386×10^{-8}	0.266137

Supplementary Table S24: *For the atrial fibrillation predictor: list of genes responsible for the top fifteen values of variance accounted for by single genes, and the corresponding variance values, both explicit and expressed as a percentage of the total predictor variance.*

Basal Cell Carcinoma

Ensembl ID	Variance Explained by Gene	% of Total Predictor Variance
ENSG00000137265	9.11032×10^{-7}	28.4835
ENSG00000125780	3.86761×10^{-7}	12.0921
ENSG00000140995	2.19694×10^{-7}	6.86874
ENSG00000177946	2.19118×10^{-7}	6.85076
ENSG00000003249	2.13993×10^{-7}	6.69052
ENSG00000179051	2.12828×10^{-7}	6.65409
ENSG00000064012	2.10488×10^{-7}	6.58092
ENSG00000155749	2.10488×10^{-7}	6.58092
ENSG00000003400	2.0926×10^{-7}	6.54255
ENSG00000114861	1.11256×10^{-7}	3.47842
ENSG00000077498	1.04526×10^{-7}	3.26803
ENSG00000205420	1.01298×10^{-7}	3.16709
ENSG00000186081	1.01298×10^{-7}	3.16709
ENSG00000268716	1.01298×10^{-7}	3.16709
ENSG00000139648	9.16756×10^{-8}	2.86625
ENSG00000158805	8.50283×10^{-8}	2.65842
ENSG00000187741	8.50283×10^{-8}	2.65842
ENSG00000101460	7.16043×10^{-8}	2.23872
ENSG00000101464	7.16043×10^{-8}	2.23872
ENSG00000196735	7.01145×10^{-8}	2.19214

Supplementary Table S25: *For the basal cell carcinoma predictor: list of genes responsible for the top fifteen values of variance accounted for by single genes, and the corresponding variance values, both explicit and expressed as a percentage of the total predictor variance.*

Breast Cancer

Ensembl ID	Variance Explained by Gene	% of Total Predictor Variance
ENSG00000066468	0.0000212362	14.7834
ENSG00000103460	0.0000200173	13.9349
ENSG00000196588	2.17644×10^{-6}	1.51512
ENSG00000120262	2.06537×10^{-6}	1.43779
ENSG00000163491	1.96784×10^{-6}	1.3699
ENSG00000074527	1.9048×10^{-6}	1.32601
ENSG00000164362	1.66297×10^{-6}	1.15766
ENSG00000140718	1.49019×10^{-6}	1.03739
ENSG00000108175	1.45587×10^{-6}	1.01349
ENSG00000166446	1.29111×10^{-6}	0.898797
ENSG00000049656	1.26148×10^{-6}	0.878169
ENSG00000138311	7.52238×10^{-7}	0.523666
ENSG00000071967	6.70661×10^{-7}	0.466876
ENSG00000167081	6.5404×10^{-7}	0.455306
ENSG00000136267	6.13165×10^{-7}	0.426851

Supplementary Table S26: *For the breast cancer predictor: list of genes responsible for the top fifteen values of variance accounted for by single genes, and the corresponding variance values, both explicit and expressed as a percentage of the total predictor variance.*

Coronary Artery Disease

Ensembl ID	Variance Explained by Gene	% of Total Predictor Variance
ENSG00000198670	0.000204929	8.14914
ENSG00000112137	0.000154524	6.14475
ENSG00000122194	0.000115362	4.58747
ENSG00000146477	0.0000584146	2.3229
ENSG00000143126	0.000056525	2.24775
ENSG00000134222	0.0000565249	2.24775
ENSG00000221986	0.0000565246	2.24774
ENSG00000091732	0.000022669	0.901448
ENSG00000130203	0.0000203651	0.809834
ENSG00000130204	0.0000203646	0.809811
ENSG00000181652	0.0000170687	0.678747
ENSG00000164867	0.0000170686	0.678746
ENSG00000055118	0.0000170678	0.678713
ENSG00000134871	0.0000158485	0.630227
ENSG00000130208	0.0000151627	0.602955

Supplementary Table S27: *For the coronary artery disease predictor: list of genes responsible for the top fifteen values of variance accounted for by single genes, and the corresponding variance values, both explicit and expressed as a percentage of the total predictor variance.*

Diabetes - Type 2

Ensembl ID	Variance Explained by Gene	% of Total Predictor Variance
ENSG00000148737	0.0000449243	24.795
ENSG00000073792	6.41139×10^{-6}	3.53863
ENSG00000053918	3.32273×10^{-6}	1.83391
ENSG00000145996	2.75949×10^{-6}	1.52304
ENSG00000145730	1.68939×10^{-6}	0.932423
ENSG00000173175	1.50627×10^{-6}	0.831356
ENSG00000115970	1.40593×10^{-6}	0.775975
ENSG00000164756	1.38849×10^{-6}	0.766351
ENSG00000029534	1.36035×10^{-6}	0.750819
ENSG00000165066	1.36035×10^{-6}	0.750815
ENSG00000196781	1.29152×10^{-6}	0.712827
ENSG00000138002	1.27887×10^{-6}	0.705844
ENSG00000115226	1.27887×10^{-6}	0.705844
ENSG00000084734	1.27887×10^{-6}	0.705844
ENSG00000233438	1.27887×10^{-6}	0.705844
ENSG00000152804	1.27043×10^{-6}	0.70119
ENSG00000108175	1.24929×10^{-6}	0.689518
ENSG00000149948	1.20786×10^{-6}	0.666652

Supplementary Table S29: *For the type-2 diabetes predictor: list of genes responsible for the top fifteen values of variance accounted for by single genes, and the corresponding variance values, both explicit and expressed as a percentage of the total predictor variance.*

Diabetes - Type 1

Ensembl ID	Variance Explained by Gene	% of Total Predictor Variance
ENSG00000196735	1.71762×10^{-6}	14.2359
ENSG00000196126	1.10313×10^{-6}	9.14287
ENSG00000179344	8.43201×10^{-7}	6.98856
ENSG00000204290	5.28293×10^{-7}	4.37856
ENSG00000116793	4.17049×10^{-7}	3.45655
ENSG00000081019	4.17049×10^{-7}	3.45655
ENSG00000129965	3.03197×10^{-7}	2.51294
ENSG00000254647	3.03197×10^{-7}	2.51294
ENSG00000180176	3.03197×10^{-7}	2.51294
ENSG00000167244	2.96451×10^{-7}	2.45702
ENSG00000237541	2.85331×10^{-7}	2.36486
ENSG00000204287	2.25475×10^{-7}	1.86877
ENSG00000148737	1.82298×10^{-7}	1.51091
ENSG00000232629	1.80438×10^{-7}	1.49549
ENSG00000204536	1.11524×10^{-7}	0.924323
ENSG00000137310	1.11524×10^{-7}	0.924323
ENSG00000204531	1.11524×10^{-7}	0.924323
ENSG00000206344	1.11524×10^{-7}	0.924323
ENSG00000204392	1.04034×10^{-7}	0.862246
ENSG00000204390	9.68184×10^{-8}	0.802444
ENSG00000204389	9.68184×10^{-8}	0.802444
ENSG00000204388	9.68184×10^{-8}	0.802444
ENSG00000204387	9.68184×10^{-8}	0.802444
ENSG00000204386	9.68184×10^{-8}	0.802444
ENSG00000204252	8.09372×10^{-8}	0.670819

Supplementary Table S28: *For the type-1 diabetes predictor: list of genes responsible for the top fifteen values of variance accounted for by single genes, and the corresponding variance values, both explicit and expressed as a percentage of the total predictor variance.*

Diastolic Blood Pressure

Ensembl ID	Variance Explained by Gene	% of Total Predictor Variance
ENSG00000205517	0.00026354	0.802109
ENSG00000111252	0.00026058	0.793101
ENSG00000204842	0.000260509	0.792884
ENSG00000198003	0.000259275	0.789127
ENSG00000130175	0.000246709	0.750883
ENSG00000168038	0.000239419	0.728695
ENSG00000123454	0.00021092	0.641956
ENSG00000123453	0.000209687	0.638203
ENSG00000138821	0.000181787	0.553286
ENSG00000138675	0.000177214	0.539366
ENSG00000177000	0.000170691	0.519515
ENSG00000011021	0.000170045	0.517549
ENSG00000165995	0.000169235	0.515084
ENSG00000215910	0.000166846	0.507812
ENSG00000182511	0.000162822	0.495565

Supplementary Table S30: *For the diastolic blood pressure predictor: list of genes responsible for the top fifteen values of variance accounted for by single genes, and the corresponding variance values, both explicit and expressed as a percentage of the total predictor variance.*

Education Years

Ensembl ID	Variance Explained by Gene	% of Total Predictor Variance
ENSG00000140945	0.000117975	0.234252
ENSG00000101489	0.000103631	0.20577
ENSG00000153310	0.0000979671	0.194524
ENSG00000179915	0.000093737	0.186125
ENSG00000145934	0.0000880685	0.174869
ENSG00000108684	0.000079856	0.158563
ENSG00000078328	0.0000798313	0.158514
ENSG00000119866	0.0000769443	0.152781
ENSG00000164061	0.0000741843	0.147301
ENSG00000188580	0.0000732577	0.145461
ENSG00000101109	0.000071637	0.142243
ENSG00000168702	0.0000694311	0.137863
ENSG00000075884	0.000068552	0.136117
ENSG00000183715	0.0000669065	0.13285
ENSG00000176171	0.0000657761	0.130606

Supplementary Table S31: *For the education years predictor: list of genes responsible for the top fifteen values of variance accounted for by single genes, and the corresponding variance values, both explicit and expressed as a percentage of the total predictor variance.*

Gallstones

Ensembl ID	Variance Explained by Gene	% of Total Predictor Variance
ENSG00000143921	0.0000278316	83.2315
ENSG00000138075	0.0000276955	82.8245
ENSG00000138036	0.0000268878	80.4092
ENSG00000152527	6.84056×10^{-7}	2.0457
ENSG00000197249	5.88871×10^{-7}	1.76105
ENSG00000105398	5.81249×10^{-7}	1.73825
ENSG00000198589	5.33802×10^{-7}	1.59636
ENSG00000170390	5.3318×10^{-7}	1.5945
ENSG00000101076	4.42043×10^{-7}	1.32195
ENSG00000005471	3.63638×10^{-7}	1.08748
ENSG00000169903	3.58726×10^{-7}	1.07279
ENSG00000085563	3.49533×10^{-7}	1.0453
ENSG00000176920	2.11116×10^{-7}	0.631352
ENSG00000176909	2.11116×10^{-7}	0.631352
ENSG00000105538	2.11116×10^{-7}	0.631352
ENSG00000182264	2.11116×10^{-7}	0.631352
ENSG00000215114	1.42874×10^{-7}	0.427272
ENSG00000167910	1.37481×10^{-7}	0.411142

Supplementary Table S32: *For the gallstones predictor: list of genes responsible for the top fifteen values of variance accounted for by single genes, and the corresponding variance values, both explicit and expressed as a percentage of the total predictor variance.*

Glaucoma

Ensembl ID	Variance Explained by Gene	% of Total Predictor Variance
ENSG00000143149	1.91058×10^{-6}	19.1727
ENSG00000143183	1.91058×10^{-6}	19.1727
ENSG00000007237	4.46775×10^{-7}	4.4834
ENSG00000264194	4.38653×10^{-7}	4.4019
ENSG00000196526	3.18499×10^{-7}	3.19614
ENSG00000264545	2.11973×10^{-7}	2.12716
ENSG00000136944	1.45785×10^{-7}	1.46296
ENSG00000077044	1.33192×10^{-7}	1.33658
ENSG00000179008	1.19163×10^{-7}	1.1958
ENSG00000184302	1.19163×10^{-7}	1.1958
ENSG00000171435	1.09544×10^{-7}	1.09927
ENSG00000147883	1.03397×10^{-7}	1.0376
ENSG00000157110	9.30046×10^{-8}	0.933304
ENSG00000183044	7.686×10^{-8}	0.771292
ENSG00000115970	7.37166×10^{-8}	0.739748
ENSG00000138193	7.18263×10^{-8}	0.720779
ENSG00000112685	6.58665×10^{-8}	0.660972

Supplementary Table S33: *For the glaucoma predictor: list of genes responsible for the top fifteen values of variance accounted for by single genes, and the corresponding variance values, both explicit and expressed as a percentage of the total predictor variance.*

Gout

Ensembl ID	Variance Explained by Gene	% of Total Predictor Variance
ENSG00000118777	0.0000202008	56.9739
ENSG00000109667	4.39332×10^{-6}	12.3908
ENSG00000138002	1.25013×10^{-6}	3.52582
ENSG00000115226	1.25013×10^{-6}	3.52582
ENSG00000084734	1.25013×10^{-6}	3.52582
ENSG00000196616	1.23783×10^{-6}	3.49115
ENSG00000248144	1.23783×10^{-6}	3.49115
ENSG00000187758	1.23775×10^{-6}	3.49093
ENSG00000233438	1.2312×10^{-6}	3.47244
ENSG00000124568	1.04791×10^{-6}	2.9555
ENSG00000124564	1.04791×10^{-6}	2.9555
ENSG00000168065	1.02306×10^{-6}	2.88542
ENSG00000197891	9.27473×10^{-7}	2.61582
ENSG00000265491	4.92238×10^{-7}	1.3883
ENSG00000174827	4.72186×10^{-7}	1.33174
ENSG00000117281	4.72186×10^{-7}	1.33174
ENSG00000165449	3.68976×10^{-7}	1.04065
ENSG00000110076	3.15776×10^{-7}	0.890607
ENSG00000179912	2.86978×10^{-7}	0.809385
ENSG00000163935	2.22875×10^{-7}	0.62859
ENSG00000272305	2.22875×10^{-7}	0.62859

Supplementary Table S34: *For the gout predictor: list of genes responsible for the top fifteen values of variance accounted for by single genes, and the corresponding variance values, both explicit and expressed as a percentage of the total predictor variance.*

Heart Attack

Ensembl ID	Variance Explained by Gene	% of Total Predictor Variance
ENSG00000198670	6.52514×10^{-6}	21.325
ENSG00000154305	1.30816×10^{-6}	4.27522
ENSG00000186063	1.30816×10^{-6}	4.27522
ENSG00000112137	9.77669×10^{-7}	3.19514
ENSG00000143126	8.24884×10^{-7}	2.69582
ENSG00000134222	8.24884×10^{-7}	2.69582
ENSG00000221986	8.24884×10^{-7}	2.69582
ENSG00000130204	6.85983×10^{-7}	2.24188
ENSG00000130203	6.85983×10^{-7}	2.24188
ENSG00000130208	6.85983×10^{-7}	2.24188
ENSG00000267467	6.69128×10^{-7}	2.18679
ENSG00000224916	6.69128×10^{-7}	2.18679
ENSG00000118526	6.56704×10^{-7}	2.14619
ENSG00000130164	6.16851×10^{-7}	2.01594
ENSG00000127616	5.64743×10^{-7}	1.84565
ENSG00000234906	5.05309×10^{-7}	1.65141
ENSG00000187498	3.40483×10^{-7}	1.11274
ENSG00000091732	3.29055×10^{-7}	1.07539
ENSG00000111252	2.99308×10^{-7}	0.978174
ENSG00000204842	2.99308×10^{-7}	0.978174
ENSG00000115970	2.8221×10^{-7}	0.922297
ENSG00000112531	1.96104×10^{-7}	0.640892

Supplementary Table S35: *For the heart attack predictor: list of genes responsible for the top fifteen values of variance accounted for by single genes, and the corresponding variance values, both explicit and expressed as a percentage of the total predictor variance.*

Height

Ensembl ID	Variance Explained by Gene	% of Total Predictor Variance
ENSG00000140470	0.00202515	0.885937
ENSG00000157766	0.00178485	0.780812
ENSG00000116183	0.00169709	0.742421
ENSG00000126001	0.0016775	0.733849
ENSG00000101019	0.00165261	0.722961
ENSG00000204183	0.00163949	0.717221
ENSG00000125965	0.00163949	0.717221
ENSG00000156218	0.00135713	0.593697
ENSG00000164161	0.0012893	0.564027
ENSG00000140443	0.00127966	0.559809
ENSG00000140511	0.00124035	0.54261
ENSG00000172201	0.00120895	0.528875
ENSG00000144535	0.00120723	0.52812
ENSG00000066827	0.00116436	0.509369
ENSG00000115380	0.00115624	0.505814
ENSG00000176124	0.00115192	0.503927

Supplementary Table S36: *For the height predictor: list of genes responsible for the top fifteen values of variance accounted for by single genes, and the corresponding variance values, both explicit and expressed as a percentage of the total predictor variance.*

High Cholesterol

Ensembl ID	Variance Explained by Gene	% of Total Predictor Variance
ENSG00000130164	0.000159927	11.0888
ENSG00000130208	0.00014979	10.3859
ENSG00000130204	0.000142489	9.87962
ENSG00000130203	0.000141593	9.81752
ENSG00000127616	0.00013607	9.43459
ENSG00000221986	0.000104152	7.22154
ENSG00000143126	0.000104132	7.22014
ENSG00000134222	0.000104132	7.22014
ENSG00000267467	0.000095527	6.62348
ENSG00000224916	0.000095527	6.62348
ENSG00000130202	0.0000908673	6.3004
ENSG00000084674	0.0000722735	5.01117
ENSG00000137656	0.0000663571	4.60095
ENSG00000109917	0.0000660421	4.57911
ENSG00000110243	0.0000660421	4.57911
ENSG00000169174	0.0000637275	4.41863
ENSG00000162402	0.0000609838	4.22839
ENSG00000162399	0.000060702	4.20885

Supplementary Table S37: *For the high cholesterol predictor: list of genes responsible for the top fifteen values of variance accounted for by single genes, and the corresponding variance values, both explicit and expressed as a percentage of the total predictor variance.*

Hypertension

Ensembl ID	Variance Explained by Gene	% of Total Predictor Variance
ENSG00000138675	0.0000739221	1.42576
ENSG00000205517	0.0000609586	1.17573
ENSG00000198003	0.0000608256	1.17317
ENSG00000130175	0.0000590379	1.13869
ENSG00000171105	0.000048139	0.928475
ENSG00000111252	0.000042734	0.824227
ENSG00000204842	0.0000427337	0.824221
ENSG00000171303	0.000040641	0.783858
ENSG00000183346	0.0000364154	0.702357
ENSG00000140564	0.0000340002	0.655775
ENSG00000182511	0.0000340002	0.655775
ENSG00000196547	0.0000338974	0.653792
ENSG00000130592	0.000030334	0.585063
ENSG00000130598	0.0000302174	0.582815
ENSG00000165995	0.0000293892	0.56684
ENSG00000138193	0.0000276911	0.534089

Supplementary Table S38: *For the hypertension predictor: list of genes responsible for the top fifteen values of variance accounted for by single genes, and the corresponding variance values, both explicit and expressed as a percentage of the total predictor variance.*

Hypothyroidism

Ensembl ID	Variance Explained by Gene	% of Total Predictor Variance
ENSG00000081019	0.0000352729	7.77261
ENSG00000134242	0.0000342818	7.55422
ENSG00000111252	0.0000295005	6.50062
ENSG00000204842	0.0000295005	6.50062
ENSG00000196735	0.0000240816	5.30654
ENSG00000179344	0.0000222207	4.89647
ENSG00000163599	0.0000145232	3.20029
ENSG00000273167	8.09495×10^{-6}	1.78378
ENSG00000182957	8.09495×10^{-6}	1.78378
ENSG00000134215	7.84936×10^{-6}	1.72966
ENSG00000112182	6.94025×10^{-6}	1.52933
ENSG00000204520	6.40646×10^{-6}	1.41171
ENSG00000145012	5.63275×10^{-6}	1.24122
ENSG00000100385	5.00989×10^{-6}	1.10396
ENSG00000138378	4.95025×10^{-6}	1.09082
ENSG00000223865	4.75552×10^{-6}	1.04791
ENSG00000231389	4.74254×10^{-6}	1.04505

Supplementary Table S39: *For the hypothyroidism predictor: list of genes responsible for the top fifteen values of variance accounted for by single genes, and the corresponding variance values, both explicit and expressed as a percentage of the total predictor variance.*

Malignant Melanoma

Ensembl ID	Variance Explained by Gene	% of Total Predictor Variance
ENSG00000198211	5.68246×10^{-7}	42.8898
ENSG00000258947	5.68246×10^{-7}	42.8898
ENSG00000141002	5.51776×10^{-7}	41.6467
ENSG00000258839	5.51776×10^{-7}	41.6467
ENSG00000140995	4.88084×10^{-7}	36.8394
ENSG00000077498	1.79479×10^{-7}	13.5467
ENSG00000158805	1.08341×10^{-7}	8.17731
ENSG00000187741	1.08341×10^{-7}	8.17731
ENSG00000164362	7.85712×10^{-8}	5.93036
ENSG00000049656	7.85712×10^{-8}	5.93036
ENSG00000151789	2.6662×10^{-8}	2.01238
ENSG00000005884	2.06455×10^{-8}	1.55827
ENSG00000005882	2.06455×10^{-8}	1.55827
ENSG00000172292	1.84488×10^{-8}	1.39247
ENSG00000177946	1.64698×10^{-8}	1.2431
ENSG00000101460	1.60221×10^{-8}	1.20931
ENSG00000101464	1.60221×10^{-8}	1.20931
ENSG00000159110	1.16468×10^{-8}	0.879072
ENSG00000249624	1.16468×10^{-8}	0.879072
ENSG00000243646	1.16468×10^{-8}	0.879072
ENSG00000139644	1.11346×10^{-8}	0.840415
ENSG00000145996	9.83099×10^{-9}	0.742018
ENSG00000150995	9.41989×10^{-9}	0.71099

Supplementary Table S40: *For the malignant melanoma predictor: list of genes responsible for the top fifteen values of variance accounted for by single genes, and the corresponding variance values, both explicit and expressed as a percentage of the total predictor variance.*

Menopause

Ensembl ID	Variance Explained by Gene	% of Total Predictor Variance
ENSG00000183878	0.339579	24.2854
ENSG00000125885	0.117767	8.42228
ENSG00000089195	0.116968	8.3651
ENSG00000127311	0.0924778	6.61366
ENSG00000160469	0.0629791	4.50402
ENSG00000180061	0.0629791	4.50402
ENSG00000133247	0.0629791	4.50402
ENSG00000160471	0.0629791	4.50402
ENSG00000267531	0.0629791	4.50402
ENSG00000187840	0.0441477	3.15727
ENSG00000163312	0.0322135	2.30379
ENSG00000163319	0.0322135	2.30379
ENSG00000163322	0.0322135	2.30379
ENSG00000087206	0.0251368	1.79769
ENSG00000113761	0.023128	1.65403
ENSG00000196531	0.017758	1.26998
ENSG00000198056	0.017758	1.26998
ENSG00000025423	0.017758	1.26998
ENSG00000151726	0.0165154	1.18112
ENSG00000158315	0.0160452	1.14749
ENSG00000116954	0.0160272	1.14621
ENSG00000214114	0.0160272	1.14621
ENSG00000274944	0.0160272	1.14621
ENSG00000131233	0.0160272	1.14621
ENSG00000155974	0.0154474	1.10474
ENSG00000234719	0.0121138	0.866332

Supplementary Table S41: *For the menopause predictor: list of genes responsible for the top fifteen values of variance accounted for by single genes, and the corresponding variance values, both explicit and expressed as a percentage of the total predictor variance.*

Pulse Rate

Ensembl ID	Variance Explained by Gene	% of Total Predictor Variance
ENSG00000092054	0.0016225	3.9998
ENSG00000197616	0.00157533	3.8835
ENSG00000163492	0.00150449	3.70887
ENSG00000166091	0.00120182	2.96274
ENSG00000166090	0.00114524	2.82326
ENSG00000155657	0.000932079	2.29777
ENSG00000149633	0.000932021	2.29762
ENSG00000100842	0.000742851	1.83128
ENSG00000196376	0.000581659	1.43391
ENSG00000182732	0.00052007	1.28208
ENSG00000165801	0.000430485	1.06123
ENSG00000165804	0.000430485	1.06123
ENSG00000232070	0.000430485	1.06123
ENSG00000165795	0.000428352	1.05598
ENSG00000173431	0.000428338	1.05594
ENSG00000174059	0.000394401	0.972278
ENSG00000089225	0.000315557	0.777911

Supplementary Table S42: *For the pulse rate predictor: list of genes responsible for the top fifteen values of variance accounted for by single genes, and the corresponding variance values, both explicit and expressed as a percentage of the total predictor variance.*

Systolic Blood Pressure

Ensembl ID	Variance Explained by Gene	% of Total Predictor Variance
ENSG00000205517	0.000244798	0.849217
ENSG00000165995	0.000244118	0.846856
ENSG00000198003	0.000241788	0.838773
ENSG00000130175	0.00023594	0.818486
ENSG00000011021	0.00020999	0.728465
ENSG00000177000	0.000203954	0.707526
ENSG00000215910	0.000203195	0.704894
ENSG00000070961	0.000200379	0.695125
ENSG00000171105	0.000196777	0.682628
ENSG00000138675	0.00019149	0.664289
ENSG00000198373	0.000156504	0.54292
ENSG00000157322	0.000156504	0.54292
ENSG00000138193	0.00014907	0.517132
ENSG00000196547	0.000140354	0.486896
ENSG00000165895	0.000137298	0.476292
ENSG00000065054	0.000124649	0.432413

Supplementary Table S43: *For the systolic blood pressure predictor: list of genes responsible for the top fifteen values of variance accounted for by single genes, and the corresponding variance values, both explicit and expressed as a percentage of the total predictor variance.*

Gallstones SNPs on DYNC2LI1

SNP ID	Variance Explained by SNP	% of Total Predictor Variance
Affx-20090007	0.0000257517	77.0116
rs17031488	6.80885×10^{-7}	2.03622
rs7599296	4.55229×10^{-7}	1.36138

Supplementary Table S44: *For the gallstones predictor: list of SNPs located on the novel DYNC2LI1 gene, and the corresponding variance values, both explicit and expressed as a percentage of the total predictor variance.*

Glaucoma SNPs on ALDH9A1

SNP ID	Variance Explained by SNP	% of Total Predictor Variance
rs4656461	1.91058×10^{-6}	19.1727

Supplementary Table S45: *For the glaucoma predictor: list of SNPs located on the novel ALDH9A1 gene, and the corresponding variance values, both explicit and expressed as a percentage of the total predictor variance.*

Malignant Melanoma SNPs on AC092143.1, TUBB3, TCF25, DEF8

SNP ID	Variance Explained by SNP	% of Total Predictor Variance
Affx-35293625	4.57025×10^{-7}	34.4951
rs11538871	8.01617×10^{-8}	6.0504
rs8063761	1.52938×10^{-8}	1.15434
rs1805008	7.82105×10^{-9}	0.590314
rs11547464	4.99642×10^{-9}	0.377118
rs1805009	1.77245×10^{-9}	0.13378
rs8049897	1.12683×10^{-9}	0.0850505
rs117204628	4.91714×10^{-11}	0.00371134

Supplementary Table S46: *For the malignant melanoma predictor: list of SNPs located on the novel AC092143.1, TUBB3, TCF25, and DEF8 genes, and the corresponding variance values, both explicit and expressed as a percentage of the total predictor variance.*

Menopause SNPs on UTY

SNP ID	Variance Explained by SNP	% of Total Predictor Variance
rs1236440	0.339579	24.2854

Supplementary Table S47: *For the menopause predictor: list of SNPs located on the novel UTY gene, and the corresponding variance values, both explicit and expressed as a percentage of the total predictor variance.*

Basal Cell Carcinoma Predictor SNPs located on IRF4 and TGM3

SNP ID	Variance Explained by SNP	% of Total Predictor Variance	Associated Gene
rs12203592	9.11032×10^{-7}	28.4835	ENSG00000137265
rs214803	3.86761×10^{-7}	12.0921	ENSG00000125780

Supplementary Table S48: *List of basal cell carcinoma predictor SNPs located on the IRF4 and TGM3 genes, and the corresponding variance values, both explicit and expressed as a percentage of the total predictor variance. The rightmost column displays the gene on which the SNP is located.*

Breast Cancer Predictor SNPs located on FGFR2 and TOX3

SNP ID	Variance Explained by SNP	% of Total Predictor Variance	Associated Gene
rs2981575	0.000020383	14.1895	ENSG00000066468
rs4784227	0.0000200051	13.9264	ENSG00000103460
rs2981579	8.2982×10^{-7}	0.577674	ENSG00000066468
rs1219648	2.33801×10^{-8}	0.0162759	ENSG00000066468
rs45512493	1.10672×10^{-8}	0.00770439	ENSG00000103460
rs3803662	7.29627×10^{-10}	0.000507925	ENSG00000103460
rs4784220	3.98991×10^{-10}	0.000277755	ENSG00000103460

Supplementary Table S49: *List of breast cancer predictor SNPs located on the FGFR2 and TOX3 genes, and the corresponding variance values, both explicit and expressed as a percentage of the total predictor variance. The rightmost column displays the gene on which the SNP is located.*

Type-2 Diabetes Predictor SNPs located on TCF7L2

SNP ID	Variance Explained by SNP	% of Total Predictor Variance	Associated Gene
rs7903146	0.000044865	24.7623	ENSG00000148737
rs34855922	5.22513×10^{-8}	0.028839	ENSG00000148737
rs11196181	3.41416×10^{-9}	0.00188437	ENSG00000148737
rs1362943	2.6939×10^{-9}	0.00148684	ENSG00000148737
rs7908486	9.94264×10^{-10}	0.000548763	ENSG00000148737

Supplementary Table S50: *List of type-2 diabetes predictor SNPs located on the TCF7L2 gene, and the corresponding variance values, both explicit and expressed as a percentage of the total predictor variance. The rightmost column displays the gene on which the SNP is located.*

Gout Predictor SNPs located on ABCG2 and SLC2A9

SNP ID	Variance Explained by SNP	% of Total Predictor Variance	Associated Gene
rs2231142	0.0000194608	54.8867	ENSG00000118777
rs3775948	2.81558×10^{-6}	7.94098	ENSG00000109667
rs16890979	7.25401×10^{-7}	2.0459	ENSG00000109667
rs3114020	6.42128×10^{-7}	1.81104	ENSG00000118777
rs16891234	4.02312×10^{-7}	1.13467	ENSG00000109667
rs13129697	2.26603×10^{-7}	0.639104	ENSG00000109667
rs73223775	1.58984×10^{-7}	0.448395	ENSG00000109667
rs3114018	7.09016×10^{-8}	0.199969	ENSG00000118777
rs737267	2.99228×10^{-8}	0.0843934	ENSG00000109667
rs34783571	2.70102×10^{-8}	0.0761788	ENSG00000118777
rs114756544	1.86542×10^{-8}	0.0526118	ENSG00000109667
rs734553	1.19146×10^{-8}	0.0336035	ENSG00000109667
rs6833878	1.95564×10^{-9}	0.00551565	ENSG00000109667
rs73225891	1.45697×10^{-9}	0.00410921	ENSG00000109667
rs3733591	5.42004×10^{-10}	0.00152865	ENSG00000109667

Supplementary Table S53: *List of gout predictor SNPs located on the ABCG2 and SLC2A9 genes, and the corresponding variance values, both explicit and expressed as a percentage of the total predictor variance. The rightmost column displays the gene on which the SNP is located.*

Gallstones Predictor SNPs located on ABCG8, ABCG5, and DYNC2LI1

SNP ID	Variance Explained by SNP	% of Total Predictor Variance	Associated Gene
Affx-20090007	0.0000257517	77.0116	ENSG00000138036
Affx-20090007	0.0000257517	77.0116	ENSG00000138075
Affx-20090007	0.0000257517	77.0116	ENSG00000143921
rs4245791	1.48854×10^{-6}	4.45153	ENSG00000138075
rs4245791	1.48854×10^{-6}	4.45153	ENSG00000143921
rs17031488	6.80885×10^{-7}	2.03622	ENSG00000138036
rs7599296	4.55229×10^{-7}	1.36138	ENSG00000138036
rs7599296	4.55229×10^{-7}	1.36138	ENSG00000138075
rs7599296	4.55229×10^{-7}	1.36138	ENSG00000143921
Affx-20090699	1.36103×10^{-7}	0.407021	ENSG00000143921

Supplementary Table S51: *List of gallstones predictor SNPs located on the ABCG8, ABCG5 and DYNC2LI1 genes, and the corresponding variance values, both explicit and expressed as a percentage of the total predictor variance. The rightmost column displays the gene on which the SNP is located.*

Glaucoma Predictor SNPs located on ALDH9A1 and TMCO1

SNP ID	Variance Explained by SNP	% of Total Predictor Variance	Associated Gene
rs4656461	1.91058×10^{-6}	19.1727	ENSG00000143149
rs4656461	1.91058×10^{-6}	19.1727	ENSG00000143183

Supplementary Table S52: *List of glaucoma predictor SNPs located on the ALDH9A1 and TMCO1 genes, and the corresponding variance values, both explicit and expressed as a percentage of the total predictor variance. The rightmost column displays the gene on which the SNP is located.*

Heart Attack Predictor SNPs located on LPA

SNP ID	Variance Explained by SNP	% of Total Predictor Variance	Associated Gene
rs10455872	4.42645×10^{-6}	14.4662	ENSG00000198670
rs117733303	2.05337×10^{-6}	6.71066	ENSG00000198670
rs73596816	2.92187×10^{-8}	0.0954903	ENSG00000198670
rs9457998	1.6105×10^{-8}	0.0526332	ENSG00000198670

Supplementary Table S54: *List of heart attack predictor SNPs located on the LPA gene, and the corresponding variance values, both explicit and expressed as a percentage of the total predictor variance. The rightmost column displays the gene on which the SNP is located.*

Malignant Melanoma Predictor SNPs located on AC092143.1, TUBB3, TCF25, MC1R and DEF8

SNP ID	Variance Explained by SNP	% of Total Predictor Variance	Associated Gene
Affx-35293625	4.57025×10^{-7}	34.4951	ENSG00000141002
Affx-35293625	4.57025×10^{-7}	34.4951	ENSG00000258839
Affx-35293625	4.57025×10^{-7}	34.4951	ENSG00000198211
Affx-35293625	4.57025×10^{-7}	34.4951	ENSG00000258947
Affx-35293625	4.57025×10^{-7}	34.4951	ENSG00000140995
rs11538871	8.01617×10^{-8}	6.0504	ENSG00000141002
rs11538871	8.01617×10^{-8}	6.0504	ENSG00000258839
rs11538871	8.01617×10^{-8}	6.0504	ENSG00000198211
rs11538871	8.01617×10^{-8}	6.0504	ENSG00000258947
rs8063761	1.52938×10^{-8}	1.15434	ENSG00000198211
rs8063761	1.52938×10^{-8}	1.15434	ENSG00000258947
rs8063761	1.52938×10^{-8}	1.15434	ENSG00000140995
rs1805008	7.82105×10^{-9}	0.590314	ENSG00000141002
rs1805008	7.82105×10^{-9}	0.590314	ENSG00000258839
rs1805008	7.82105×10^{-9}	0.590314	ENSG00000198211
rs1805008	7.82105×10^{-9}	0.590314	ENSG00000258947
rs1805008	7.82105×10^{-9}	0.590314	ENSG00000140995
rs11547464	4.99642×10^{-9}	0.377118	ENSG00000141002
rs11547464	4.99642×10^{-9}	0.377118	ENSG00000258839
rs11547464	4.99642×10^{-9}	0.377118	ENSG00000198211
rs11547464	4.99642×10^{-9}	0.377118	ENSG00000258947
rs11547464	4.99642×10^{-9}	0.377118	ENSG00000140995
rs1805009	1.77245×10^{-9}	0.13378	ENSG00000141002
rs1805009	1.77245×10^{-9}	0.13378	ENSG00000258839
rs1805009	1.77245×10^{-9}	0.13378	ENSG00000198211
rs1805009	1.77245×10^{-9}	0.13378	ENSG00000258947
rs1805009	1.77245×10^{-9}	0.13378	ENSG00000140995
rs8049897	1.12683×10^{-9}	0.0850505	ENSG00000198211
rs8049897	1.12683×10^{-9}	0.0850505	ENSG00000258947
rs8049897	1.12683×10^{-9}	0.0850505	ENSG00000140995
rs117204628	4.91714×10^{-11}	0.00371134	ENSG00000198211
rs117204628	4.91714×10^{-11}	0.00371134	ENSG00000258947
rs117204628	4.91714×10^{-11}	0.00371134	ENSG00000140995

Supplementary Table S55: *List of malignant melanoma predictor SNPs located on the AC092143.1, TUBB3, TCF25, MC1R and DEF8 genes, and the corresponding variance values, both explicit and expressed as a percentage of the total predictor variance. The rightmost column displays the gene on which the SNP is located.*

Menopause Predictor SNPs located on UTY

SNP ID	Variance Explained by SNP	% of Total Predictor Variance	Associated Gene
rs1236440	0.339579	24.2854	ENSG00000183878

Supplementary Table S56: *List of menopause predictor SNPs located on the UTY gene, and the corresponding variance values, both explicit and expressed as a percentage of the total predictor variance. The rightmost column displays the gene on which the SNP is located.*

Appendix C: Supplementary Methods

UK Biobank Array Sequencing Data

Predictors (with the exception of the CAD predictor from [1]) were originally trained using the 2018 release of the UK Biobank data. Predictor training was restricted to genetically British individuals (as defined using ancestry principal component analysis performed by UK Biobank) [2, 3]. In 2018, the UK Biobank re-released the dataset representing approximately 500,000 individuals genotyped on two Affymetrix platforms - approximately 50,000 samples on the UKB BiLEVE Axiom array and the remainder on the UKB Biobank Axiom array. The genotype information was collected for 488,377 individuals, and 805,426 SNPs, which were then subsequently imputed to a much larger number of SNPs. More details about the design of the array can be found on these documents from the UK Biobank website: an Axiom array content summary: `http://www.ukbiobank.ac.uk/wp-content/uploads/2014/04/UK-Biobank-Axiom-Array-Content-Summary-2014-1.pdf`, and the document detailing the Axiom array: `http://www.ukbiobank.ac.uk/wp-content/uploads/2014/04/UK-Biobank-Axiom-Array-Datasheet-2014-1.pdf`. Further information about the genotyping and phenotyping used to build the original predictors can be found in [1, 4].

UK Biobank Exome Sequencing Data

In March 2019, the UK Biobank released whole-exome sequencing (WES) data for 49,960 participants [5]. Selection of participants for the study prioritized individuals with whole-body MRI imaging data from the UK Biobank Imaging Study, enhanced baseline measurements, hospital episode statistics (HES), linked primary

Phenotype	# Case Train	# Control Train	# Case Val	# Control Val		# Active
asthma	46,692	354,153	500	500		13,386
atrial fibrillation	2,939	397,906	500	500		207
basal cell carcinoma	3,624	397,221	500	500		116
breast cancer	8977	208,716	200	200		1,805
type-1 diabetes	11,006	390,439	500	500		3,175
type-2 diabetes	2,223	398,622	500	500		3,479
gallstones	17,210	394,409	500	500		274
glaucoma	3,991	396,854	500	500		1,385
gout	541	395,444	500	500		766
heart attack	8,960	391,885	500	500		1,433
high cholesterol	51,405	349,440	500	500		6,390
hypertension	106,287	294,558	500	500		17,753
hypothyroidism	19,816	381,029	500	500		5,953

Supplementary Table S57: *The number of cases and controls used in the training and validation sets for case-control phenotypes, and the sizes of the resultant active sets.*

care records, and admission to hospital with a primary diagnosis of asthma. In regards to age, sex and ancestry, the sequenced individuals are representative of the overall UK Biobank cohort. The sample set has 194 parent-offspring pairs, including 26 mother-father-child trios, 613 full-sibling pairs, 1 monozygotic twin pair and 195 second-degree genetically determined relationships.

Exomes were captured using a version of the IDT xGen Exome Research Panel v1.0. Multiplexed samples were sequenced with dual-indexed 75 x 75 bp paired-end reads on the Illumina NovaSeq 6000 platform using S2 flow cells. The specific genomic regions targeted for sequencing covered 39 megabases of the human genome, corresponding to 19,396 genes. In addition, the regions measuring 100 bp and located directly upstream and downstream of each target region were also sequenced.

A total of 4,735,722 variants located in targeted regions were identified. With adjacent (non-targeted) 100 bp regions included in the tally, a total of 9,693,526 indel and single nucleotide variants (SNVs) were observed. While only the target regions are required to meet all sequencing quality standards such as unique read coverage, variants in both target and adjacent regions were subjected to the same variant quality control metrics. Approximately 14% of coding variants identified via whole-exome sequencing were observed in the imputed sequence of 49,797 participants with both whole-exome sequencing and imputed data. 22.6% of the coding variants in the imputed data were not observed in the whole-exome sequencing data.

About Our Predictors

Predictors were derived as follows: For every phenotype considered, a small subset of the genetically British grouping of UK Biobank participants was set aside for validation, and then the predictors were trained on a large remaining subset - see Supplementary Tables S57 and S58 for the exact training and validation set sizes. Cross-validation was performed several times, and one predictor was randomly selected as the representative predictor for that particular phenotype. Each predictor consists of a set of SNP ID's, weights, effect alleles, and a value of the penalization parameter λ. True out-of-sample testing and adjacent ancestry testing was performed for many of these predictors in [4, 6]. More information can be found in the Methods section of the main text, and in [4, 6].

As described in the Methods section of the main text, the L1-penalized LASSO regression is not expected to output weights such that $X \cdot \beta$ is of order 1. However, we can still record what the total value of variance, $\sum 2\beta_i^2 f_i(1-f_i)$, is for each predictor (Supplementary Table S59).

Phenotype	# Train	# Control		# Active
diastolic blood pressure	428,981	5,000		18,383
education years	450,637	5,000		19,498
height	416,452	39,787		25,829
menopause	115,847	5,000		6,170
pulse rate	428,981	5,000		16,941
systolic blood pressure	428,977	5,000		17,097

Supplementary Table S58: *The sizes of the training and control sets for continuous phenotypes, and the sizes of the resultant active sets.*

Phenotype	$\sum_i 2\beta_i^2 f_i(1-f_i)$
asthma	0.0016
atrial fibrillation	58e-7
basal cell carcinoma	32e-7
breast cancer	14e-5
coronary artery disease	0.0025
type-1 diabetes	12e-6
type-2 diabetes	18e-5
diastolic blood pressure	0.033
education years	0.05
gallstones	33e-6
glaucoma	1e-5
gout	35e-6
heart attack	31e-6
height	0.23
high cholesterol	0.0014
hypertension	0.0052
hypothyroidism	45e-5
malignant melanoma	13e-7
menopause	1.4
pulse rate	0.041
systolic blood pressure	0.029

Supplementary Table S59: *Values of total predictor variance, $\sum_i 2\beta_i^2 f_i(1-f_i)$, for each phenotype that we consider in this paper. Each value is rounded to two significant figures.*

Appendix D: Supplementary Note

SNP-Based Heritability and Genetic Correlations of Phenotypes

Supplementary Table S60 displays a list of previously published estimates of the SNP-based heritability calculated using REML methods, for each phenotype that we consider.

The Neale lab has produced a large, ongoing analysis of heritability, genetic correlations, and GWAS analysis of thousands of UK Biobank phenotypes [22]. Using the heritability and genetic correlation browser from [22], we quote the SNP-based heritability and genetic correlations calculated by them for our selection of phenotypes in Supplementary Table S61 and Supplementary Figures S62 and S63.

The Neale group uses LD Score regression [23] to estimate heritability. It is stressed that this method of estimating heritability is very rough, and the authors hint that other methods, like REML or linear mixed models, might give more precise estimates. (These other models are also very sensitive to SNP content, sample sizes, and biases [24–31]). For this reason, we only quote the genetic heritability central value, h_g^2, and the p-value associated with whether any genetic heritability has been measured. To reproduce these results, find standard errors, sample sizes, etc. - see [22].

Phenotype	Published Values of h_g^2 Estimated Using REML Methods
asthma	0.152(0.018)[7]; 0.264(0.067)[8]
atrial fibrillation	0.096(0.012)[9]; 0.221(0.064)[10]; 0.238(0.025)[11]
basal cell carcinoma	N/A
breast cancer	0.117(0.051)[8]; 0.13(0.13)[12]
CAD	0.092(0.015)[7]; 0.159(0.002)[13]; 0.146(0.017)[8]; 0.216(0.016)[11]
type-1 diabetes	0.32(0.04)[14]
type-2 diabetes	0.297(0.022)[7]; 0.073(0.002)[13]; 0.254(0.041)[8]; 0.51(0.065)[15]
diastolic blood pressure	0.197(0.005)[11]
education years	0.194(0.002)[13]
gallstones	N/A
glaucoma	0.42(0.09)[16]
gout	0.236(0.160)[17]; 0.27(0.04)[18]
heart attack	0.190(0.025)[11]; 0.41(0.067)[15]
height	0.578(0.002)[13]; 0.50(0.18)[19]; 0.46(0.05)[19]; 0.45(0.08)[20]; 0.399(0.017)[8]; 0.614(0.004)[11]
high cholesterol	N/A
hypertension	0.255(0.014)[7]; 0.310(0.008)[11]
hypothyroidism	0.086(0.002)[13]
malignant melanoma	0.19(0.18)[12]; 0.3(0.2)[12]
menopause	0.136(0.053)[8]
pulse rate	0.13(0.01)[21]
systolic blood pressure	0.198(0.005)[11]; 0.24(0.05)[19]

Supplementary Table S60: *Previously published estimates of the SNP-based heritability (with the standard error in parentheses and corresponding citation in square brackets directly following each estimate) calculated using REML methods, for each phenotype that we consider. All genetic data sets used were restricted to participants with majority European or British ancestry.*

Phenotype	h_g^2	p-value	Phenotype	h_g^2	p-value
asthma	0.170	2.81e-36	height	0.485	6.14e-110
atrial fibrillation	0.144	1.27e-8	high cholesterol	0.126	3.22e-25
basal cell carcinoma	0.178	4.02e-6	hypertension	0.238	4.24e-116
breast cancer	0.144	8.15e-09	hypothyroidism	0.232	1.58e-22
diastolic blood pressure	0.143	1.10e-135	malignant melanoma	0.127	0.00296
education Years	0.105	1.15e-108	menopause	0.115	1.41e-19
gallstones	0.0867	3.67e-5	pulse rate	0.143	2.16e-25
glaucoma	0.128	1.90e-5	systolic blood pressure	0.151	4.93e-126
gout	0.248	1.09e-4	type-1 diabetes	0.219	0.0225
heart attack	0.144	7.06e-13	type-2 diabetes	0.123	0.00274

Supplementary Table S61: *SNP-based heritability estimates, h_g^2, and the corresponding p-values, calculated using LD score regression in [22].*

	menopause	high cholesterol	height	hypothyroidism	heart attack	pulse rate	asthma	diastolic bp	hypertension	systolic bp	edu years	atrial fibrillation
menopause	1	−0.08	0.02	−0.05	−0.11	−0.04	−0.11	−0.06	−0.08	−0.02	0.19	0.05
high cholesterol	−0.08	1	−0.2	0.17	0.6	0.13	0.09	0.37	0.57	0.42	−0.26	0.02
height	0.02	−0.2	1	0.01	−0.18	−0.08	−0.08	−0.12	−0.12	−0.15	0.23	0.24
hypothyroidism	−0.05	0.17	0.01	1	0.18	−0.05	0.07	0.05	0.1	0.01	−0.12	0.02
heart attack	−0.11	0.6	−0.18	0.18	1	0.13	0.13	0.34	0.51	0.35	−0.38	0.17
pulse rate	−0.04	0.13	−0.08	−0.05	0.13	1	0.08	0.29	0.11	0.03	−0.09	−0.03
asthma	−0.11	0.09	−0.08	0.07	0.13	0.08	1	0.07	0.11	0.06	−0.14	0.02
diastolic bp	−0.06	0.37	−0.12	0.05	0.34	0.29	0.07	1	0.78	0.67	−0.24	0.15
hypertension	−0.08	0.57	−0.12	0.1	0.51	0.11	0.11	0.78	1	0.79	−0.29	0.17
systolic bp	−0.02	0.42	−0.15	0.01	0.35	0.03	0.06	0.67	0.79	1	−0.21	0.16
edu years	0.19	−0.26	0.23	−0.12	−0.38	−0.09	−0.14	−0.24	−0.29	−0.21	1	−0.02
atrial fibrillation	0.05	0.02	0.24	0.02	0.17	−0.03	0.02	0.15	0.17	0.16	−0.02	1

Supplementary Figure S62: *Genetic correlation estimates for some of the phenotypes examined in this paper. Results are quoted from [22]. Notable overlaps are: heart attack and high cholesterol at .6, hypertension and high cholesterol at .57, hypertension and heart attack at .51, systolic blood pressure and hypertension at .79, hypertension and diastolic blood pressure at .78, systolic blood pressure and diastolic blood pressure at .67, and diastolic blood pressure and pulse rate at .29.*

	menopause	high cholesterol	height	hypothyroidism	heart attack	pulse rate	asthma	diastolic bp	hypertension	systolic bp	edu years	atrial fibrillation
menopause	−100	−1.4	−0.5	−0.57	−1.7	−0.62	−2.4	−1.4	−2.2	−0.28	−9.3	−0.54
high cholesterol	−1.4	−100	−23.	−4.5	−35.	−2.9	−2.4	−27.	−67.	−37.	−14.	−0.15
height	−0.5	−23.	−100	−0.092	−8.8	−3.5	−4.4	−10.	−9.6	−16.	−47.	−13.
hypothyroidism	−0.57	−4.5	−0.092	−100	−3.2	−0.72	−1.1	−0.61	−1.9	−0.11	−3.7	−0.22
heart attack	−1.7	−35.	−8.8	−3.2	−100	−2.6	−3.	−16.	−47.	−17.	−20.	−2.1
pulse rate	−0.62	−2.9	−3.5	−0.72	−2.6	−100	−2.2	−32.	−5.7	−0.6	−2.5	−0.25
asthma	−2.4	−2.4	−4.4	−1.1	−3.	−2.2	−100	−2.4	−4.2	−1.7	−7.6	−0.19
diastolic bp	−1.4	−27.	−10.	−0.61	−16.	−32.	−2.4	−100	−100	−100	−31.	−4.5
hypertension	−2.2	−67.	−9.6	−1.9	−47.	−5.7	−4.2	−100	−100	−100	−38.	−6.1
systolic bp	−0.28	−37.	−16.	−0.11	−17.	−0.6	−1.7	−100	−100	−100	−24.	−4.1
edu years	−9.3	−14.	−47.	−3.7	−20.	−2.5	−7.6	−31.	−38.	−24.	−100	−0.19
atrial fibrillation	−0.54	−0.15	−13.	−0.22	−2.1	−0.25	−0.19	−4.5	−6.1	−4.1	−0.19	−100

Supplementary Figure S63: *Log_{10}(p-value) corresponding to the genetic correlation results cited in Supplementary Figure S62. Values of -100 correspond to p-values that are 0 to within floating precision. All values are quoted from [22].*

References

1. Khera, A. V. *et al.* Genome-wide polygenic scores for common diseases identify individuals with risk equivalent to monogenic mutations. *Nature genetics* **50,** 1219 (2018) (cit. on p. 20).
2. Sudlow, C. *et al.* UK biobank: an open access resource for identifying the causes of a wide range of complex diseases of middle and old age. *PLoS medicine* **12,** 3 (2015) (cit. on p. 20).
3. Bycroft, C., Freeman, C. & Petkova, D. The UK Biobank resource with deep phenotyping and genomic data. *Nature* **562,** 203–209 (cit. on p. 20).

4. Lello, L., Raben, T. G., Yong, S. Y., Tellier, L. C. & Hsu, S. D. H. Genomic prediction of 16 complex disease risks including heart attack, diabetes, breast and prostate cancer. *Sci Rep* **9,** 2019 (2019) (cit. on pp. 20, 21).
5. Van Hout, C. V. *et al.* Whole exome sequencing and characterization of coding variation in 49,960 individuals in the UK Biobank. *bioRxiv* (2019) (cit. on p. 20).
6. Lello, L. *et al.* Accurate genomic prediction of human height. *Genetics* **210,** 477–497 (2018) (cit. on p. 21).
7. Loh, P.-R. *et al.* Contrasting genetic architectures of schizophrenia and other complex diseases using fast variance-components analysis. *Nature genetics* **47,** 1385 (2015) (cit. on p. 24).
8. Zaitlen, N. *et al.* Using extended genealogy to estimate components of heritability for 23 quantitative and dichotomous traits. *PLoS genetics* **9** (2013) (cit. on p. 24).
9. Nielsen, J. B. *et al.* Genome-wide study of atrial fibrillation identifies seven risk loci and highlights biological pathways and regulatory elements involved in cardiac development. *The American Journal of Human Genetics* **102,** 103–115 (2018) (cit. on p. 24).
10. Weng, L.-C. *et al.* Heritability of atrial fibrillation. *Circulation: Cardiovascular Genetics* **10,** e001838 (2017) (cit. on p. 24).
11. Verweij, N., Eppinga, R. N., Hagemeijer, Y. & van der Harst, P. Identification of 15 novel risk loci for coronary artery disease and genetic risk of recurrent events, atrial fibrillation and heart failure. *Scientific reports* **7,** 1–9 (2017) (cit. on p. 24).
12. Lu, Y. *et al.* Most common 'sporadic'cancers have a significant germline genetic component. *Human molecular genetics* **23,** 6112–6118 (2014) (cit. on p. 24).
13. Loh, P.-R., Kichaev, G., Gazal, S., Schoech, A. P. & Price, A. L. Mixed-model association for biobank-scale datasets. *Nature genetics* **50,** 906–908 (2018) (cit. on p. 24).
14. Lee, S. H., Wray, N. R., Goddard, M. E. & Visscher, P. M. Estimating missing heritability for disease from genome-wide association studies. *The American Journal of Human Genetics* **88,** 294–305 (2011) (cit. on p. 24).
15. Stahl, E. A. *et al.* Bayesian inference analyses of the polygenic architecture of rheumatoid arthritis. *Nature genetics* **44,** 483 (2012) (cit. on p. 24).
16. Cuellar-Partida, G. *et al.* Assessment of polygenic effects links primary open-angle glaucoma and age-related macular degeneration. *Scientific reports* **6,** 1–6 (2016) (cit. on p. 24).
17. Cadzow, M., Merriman, T. R. & Dalbeth, N. Performance of gout definitions for genetic epidemiological studies: analysis of UK Biobank. *Arthritis research & therapy* **19,** 181 (2017) (cit. on p. 24).
18. Köttgen, A. *et al.* Genome-wide association analyses identify 18 new loci associated with serum urate concentrations. *Nature genetics* **45,** 145–154 (2013) (cit. on p. 24).
19. Vattikuti, S., Guo, J. & Chow, C. C. Heritability and genetic correlations explained by common SNPs for metabolic syndrome traits. *PLoS genetics* **8** (2012) (cit. on p. 24).
20. Yang, J. *et al.* Common SNPs explain a large proportion of the heritability for human height. *Nature genetics* **42,** 565 (2010) (cit. on p. 24).
21. Speed, D., Holmes, J. & Balding, D. J. Evaluating and improving heritability models using summary statistics. *Nature Genetics* **52,** 458–462 (2020) (cit. on p. 24).
22. *MS Windows NT Kernel Description* `http://www.nealelab.is/uk-biobank/`. Accessed: 2020-05-23 (cit. on pp. 23–26).
23. Bulik-Sullivan, B. K. *et al.* LD Score regression distinguishes confounding from polygenicity in genome-wide association studies. *Nature genetics* **47,** 291 (2015) (cit. on p. 23).
24. Schreck, N., Piepho, H.-P. & Schlather, M. Best prediction of the additive genomic variance in random-effects models. *Genetics* **213,** 379–394 (2019) (cit. on p. 23).
25. Speed, D. *et al.* Reevaluation of SNP heritability in complex human traits. *Nature genetics* **49,** 986 (2017) (cit. on p. 23).

26. Lee, J. J. & Chow, C. C. Conditions for the validity of SNP-based heritability estimation. *Human genetics* **133,** 1011–1022 (2014) (cit. on p. 23).

27. De los Campos, G., Sorensen, D. & Gianola, D. Genomic heritability: what is it? *PLoS Genetics* **11** (2015) (cit. on p. 23).

28. Yang, J., Lee, S. H., Wray, N. R., Goddard, M. E. & Visscher, P. M. GCTA-GREML accounts for linkage disequilibrium when estimating genetic variance from genome-wide SNPs. *Proceedings of the National Academy of Sciences* **113,** E4579–E4580 (2016) (cit. on p. 23).

29. Kumar, S. K., Feldman, M. W., Rehkopf, D. H. & Tuljapurkar, S. Response to Commentary on" Limitations of GCTA as a solution to the missing heritability problem". *BioRxiv,* 039594 (2016) (cit. on p. 23).

30. Kumar, S. K., Feldman, M. W., Rehkopf, D. H. & Tuljapurkar, S. Limitations of GCTA as a solution to the missing heritability problem. *Proceedings of the National Academy of Sciences* **113,** E61–E70 (2016) (cit. on p. 23).

31. Gamazon, E. R. & Park, D. S. SNP-based heritability estimation: measurement noise, population stratification and stability. *BioRxiv,* 040055 (2016) (cit. on p. 23).
